# Response shift results of quantitative research using patient-reported outcome measures: a descriptive systematic review

**DOI:** 10.1007/s11136-023-03495-x

**Published:** 2023-09-13

**Authors:** Richard Sawatzky, Tolulope T. Sajobi, Lara Russell, Oluwagbohunmi A. Awosoga, Ayoola Ademola, Jan R. Böhnke, Oluwaseyi Lawal, Anita Brobbey, Lisa M. Lix, Amelie Anota, Véronique Sebille, Mirjam A. G. Sprangers, Mathilde G. E. Verdam, The Response Shift – in Sync Working Group

**Affiliations:** 1 School of Nursing, Trinity Western University, 22500 University Drive, Langley, BC V2Y 1Y1 Canada; 2 Centre for Advancing Health Outcomes, St. Paul’s Hospital, Vancouver, Canada; 3 University of Gothenburg Centre for Person‑Centred Care (GPCC), Sahlgrenska Academy, University of Gothenburg, Gothenburg, Sweden; 4 Department of Community Health Sciences, University of Calgary, Calgary, Canada; 5 Faculty of Health Sciences, University of Lethbridge, Lethbridge, Canada; 6 School of Health Sciences, University of Dundee, Dundee, UK; 7 Department of Community Health Sciences, University of Manitoba, Winnipeg, Canada; 8 Methodology and Quality of Life Unit in Oncology, University Hospital of Besançon, Besançon, France; 9 INSERM, MethodS in Patient-Centered Outcomes and HEalth ResEarch, SPHERE, Nantes Université, Université de Tours, CHU Nantes, 44000 Nantes, France; 10 Medical Psychology, Amsterdam UMC Location University of Amsterdam, Amsterdam, The Netherlands; 11 Mental Health, Amsterdam Public Health, Amsterdam, The Netherlands; 12 Department of Methodology and Statistics, Institute of Psychology, Leiden University, Leiden, The Netherlands

**Keywords:** Response shift, Patient-reported outcomes, Systematic review, Prevalence, Effect sizes

## Abstract

**Purpose:**

The objective of this systematic review was to describe the prevalence and magnitude of response shift effects, for different response shift methods, populations, study designs, and patient-reported outcome measures (PROM)s.

**Methods:**

A literature search was performed in MEDLINE, PSYCINFO, CINAHL, EMBASE, Social Science Citation Index, and Dissertations & Theses Global to identify longitudinal quantitative studies that examined response shift using PROMs, published before 2021. The magnitude of each response shift effect (effect sizes, R-squared or percentage of respondents with response shift) was ascertained based on reported statistical information or as stated in the manuscript. Prevalence and magnitudes of response shift effects were summarized at two levels of analysis (study and effect levels), for recalibration and reprioritization/reconceptualization separately, and for different response shift methods, and population, study design, and PROM characteristics. Analyses were conducted twice: (a) including all studies and samples, and (b) including only unrelated studies and independent samples.

**Results:**

Of the 150 included studies, 130 (86.7%) detected response shift effects. Of the 4868 effects investigated, 793 (16.3%) revealed response shift. Effect sizes could be determined for 105 (70.0%) of the studies for a total of 1130 effects, of which 537 (47.5%) resulted in detection of response shift. Whereas effect sizes varied widely, most median recalibration effect sizes (Cohen’s *d*) were between 0.20 and 0.30 and median reprioritization/reconceptualization effect sizes rarely exceeded 0.15, across the characteristics. Similar results were obtained from unrelated studies.

**Conclusion:**

The results draw attention to the need to focus on understanding variability in response shift results: Who experience response shifts, to what extent, and under which circumstances?

**Supplementary Information:**

The online version contains supplementary material available at 10.1007/s11136-023-03495-x.

## Background

**Fig. 1:**
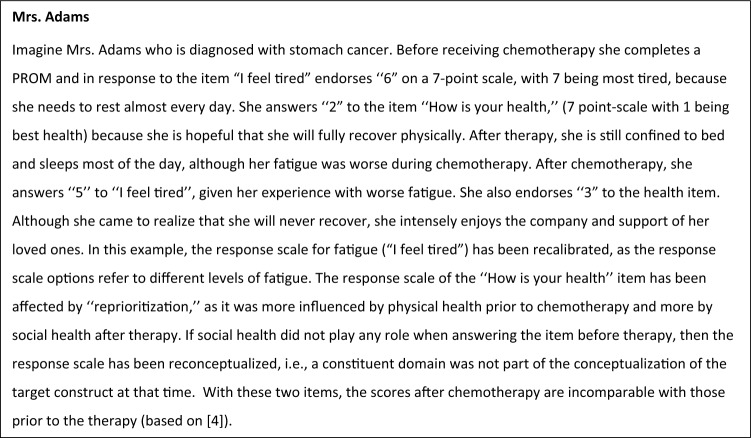
Vignette illustrating recalibration, reprioritization, and reconceptualization response shifts

Longitudinal measurements of patient-reported outcomes (PROs) can be affected by response shift. Whereas several definitions of response shift exist [1], they all draw upon the working definition provided by Sprangers and Schwartz in 1999 [2, 3], where response shift refers to a change in the meaning of one’s self-evaluation of a target construct as a result of (a) a change in the respondent’s internal standards of measurement (i.e., recalibration); (b) a change in the importance of component domains constituting the target construct (i.e., reprioritization); or (c) a redefinition of the target construct (i.e., reconceptualization). When response shift occurs, the responses to a PROM at one point in time do not have the same meaning as the responses to that PROM at another point in time (see illustrative vignette in Fig. 1, based on [4]; also see [5]). Response shift has important implications when inferences, actions and decisions in health care are made based on the use of PROMs to measure change in QOL [6]. However, despite a proliferation of research on response shift spanning several decades, a comprehensive descriptive synthesis of quantitative response shift results has thus far not been reported.

There are many different statistical methods for detecting and quantifying the magnitudes of response shift effects. Informed by our previous work [7–9], we a priori classified the methods broadly as follows: design-based methods, latent-variable methods, and regression-based methods (Table 1 for detailed descriptions and explanations). Design-based methods involve collection of data for the specific purpose of detecting response shift effects. Common examples include the then-test and individualized methods. Latent-variable methods allow for testing response shift effects by longitudinally examining the consistency (or invariance) of measurement models (e.g., structural equation models or item response theory or Rasch models). Regression-based methods involve the use of various regression analytical techniques to classify people or test for hypothesized response shift effects. Sébille et al. [8] have shown that all these methods operationalize the working definition of Sprangers and Schwartz [2, 3], albeit in different ways.

**Table 1:** Overview of response shift methods including response shift detection and effect size estimation (if possible)

Name and description	Detection of response shift	Effect size metrics as reported in the included studies
1. **Design-based methods** This family of methods requires changes or extensions to common study designs to enable the detection of response shift [3].		
1.1 Then-test The then-test is an additional measurement at follow-up occasion. Respondents complete the same measure as they did at baseline and follow-up, but now with the instruction to re-evaluate their level of baseline functioning [8, 32]. This includes: 1.1.1 *Then-test: original* Conventional use of the then-test as described above 1.1.2 *Then-test: derivative* Study-specific adaptations of the then-test. For example, when applied to valuation of health states, respondents at follow-up are asked to evaluate their own health state of that moment and are asked to recall their valuation of their health state at the previous interview [33].	*Then-test: original* Recalibration: Comparison of the mean difference between baseline and then-test. *Explanation*: For example, when chemotherapy induces fatigue, patients may adapt to this higher fatigue level. As a consequence, they may recalibrate the response scale for fatigue. This is indicated when respondents retrospectively (at the then-test) report less fatigue than they did at baseline. The comparison of the mean difference between baseline and then-test is then indicative of recalibration. *Then-test: derivative* Recalibration: Unique for each study.	*Then-test: original* Standardized mean differences (SMDs) between baseline (${\overline{X} }_{2})$ and then-test (${\overline{X} }_{1})$ scores were calculated based on available information.^a^ We used reported SMDs, if provided, when insufficient information was available to calculate the SMDs. *Then-test: derivative* Same as Then-test original.
1.2 Individualized methods This family of methods either have respondent-generated content in terms of the domains or the scale anchors are respondent generated or defined. A necessary component of individualized approaches is some respondent-generated content (e.g., items, scale anchors).		
1.2.1 *Schedule for the Evaluation of Individual Quality of Life (SEIQoL)* Respondents nominate the five most relevant domains (also called cues) to their HRQoL and assess their current functioning for each domain using a visual analogue scale (VAS) ranging from best to worst possible functioning. Patients then rank the relative importance of each domain by allocating 100 points to the five domains, using a pie chart disk (judgment analysis can also be used). The SEIQoL generates an overall index score, which is the sum of all five domain products (multiplication of each domain’s weight by its corresponding level) [34]. If the SEIQoL is administered at two points in time, response shift can be assessed [8].	Recalibration: Cannot be detected (only in combination with another method, e.g., the then-test) (e.g., [34]). Reprioritization: Statistical test of change in the domain weights. This may entail the difference in intra-class correlation coefficients between domain weights over time [35]; or subtraction of weights at follow-up from weights at baseline within unchanged domains [36]; or change in cue weights [37]. Reconceptualization: Statistical test of change in the number or type of the nominated domains over time. This may entail a mere count of domains mentioned at follow-up but not at baseline [36, 38], or the number (or percentage of) participants who changed at least one domain over time [37] or the most important domain over time [39]. A qualitative review of change in domain content is part of the procedure [8].	Recalibration, reprioritization, and reconceptualization: Effect sizes were not reported and could not be calculated based on information reported in the included studies, except for one study where the then-test method was applied to calculate SMDs based on the SEIQoL [34].
1.2.2 *Patient-Generated Index (PGI)* Respondents nominate up to five areas in relation to their disease that impacted their QOL and one additional area not related to their disease. Respondents are then asked to rate the severity of the nominated areas on a scale of 0–10 (e.g., with 0 being severe or worst they can imagine) and 10 being mild or as they would like to be). Finally, respondents are asked to distribute 12 tokens among the nominated areas, at least one to each area, and give more tokens to the areas that are in most need of improvement. The total score is calculated by multiplying the severity score by the proportion of the 12 tokens assigned to each area and then summing this across the six areas (five disease related and one non-disease related) [40]. An area-weighted score can also be calculated [41]. If the PGI is administered at two points in time, response shift can be assessed [42].	Recalibration: Cannot be detected (only in combination with another method, e.g., the then-test). Reprioritization: Statistical test of change in the domain weights (change in number of tokens) over time. This may be combined with qualitative interviews to results (e.g., [41, 43]). Reconceptualization: Statistical test of change in the number or type of areas nominated over time. This includes expansion, reduction, or completely different domains. This may involve calculating an index of change score [42] or be combined with qualitative interview results (e.g., [41, 43]).	Recalibration, Reprioritization, and Reconceptualization: Effect sizes were not reported and could not be calculated based on information reported in the included studies.
1.2.3 *Cantril’s ladder and/or changes in anchors* Respondents are asked to describe their best and worst imaginable life satisfaction as anchors for the ladder. They then rate their current life satisfaction on that ladder with the lowest rung being the worst and the highest rung being the best. If Cantril’s ladder is administered at two points in time, response shift can be assessed [3]. In some studies, patients are then invited to locate the pre-test anchors on the post-test scale with the post-test anchors indicating numbers 1 and 10. This rating scale is extended at both extremes allowing the anchor descriptions of the first assessment to be worse, better, or correspond with those of the second assessment [44, 45].	Recalibration: Statistical test of the difference between baseline and transformed baseline. The transformed scores are a function of the baseline scores and the position of the best and worse anchors in Cantril’s ladder at follow-up [44].	Recalibration: SMDs between baseline and transformed baseline scores [45] (based on the same formulas as for the then-test) or SDMs with a different denominator than provided in footnote 1 qualifying as another metric [44].
1.3 Other design-based methods		
1.3.1 *Ideal-scale approach* Respondents are asked to complete a questionnaire twice: First in reference to their actual status (e.g., how they perceive their current QOL) and second to their ideal status, e.g., how they would like their QOL to be or how they expect their QoL to change [46]. These two questionnaires are administered subsequently at the same assessment point. Administration of these two questionnaires is repeated over time [3].	Recalibration: The response scale of what ideal entails may undergo recalibration, which is indicated by a statistical test of mean changes in ideal scores over time [3]. Alternatively, change in internal standards can be captured by comparing actual and ideal status (e.g., QoL expectancies) between baseline and follow-up [46].	Recalibration: Effect sizes were not reported and could not be calculated based on information reported in the included studies.
1.3.2 *Appraisal* Changes in cognitive appraisal can be operationalized by the repeated administration of the QoL Appraisal Profile (QOLAP), QOLAP version 2 or the Brief Appraisal Profile [1, 47]. For detection of response shift, an additional method is needed, e.g., regression analysis.	Direct response shift effects: How much changes in appraisal explain the discrepancy between expected and observed QoL (e.g., residuals in a regression model reflecting unexplained variance). Moderated response shift effects: Interaction effects between appraisal change scores*catalyst [8]. No distinction is made between response shift type.	Direct and moderated response shift effects: Effect sizes were not reported and could not be calculated based on information reported in the included study.
1.3.3 *Change in importance ratings* Respondents are asked to indicate the importance of QoL domains over time, using response scales per domain (e.g., from very unimportant to very important) or by ranking the domains according to importance [48].	Reprioritization: If the relative importance of (the QoL) domains changes over time, this is indicated by statistical tests of mean change in importance ratings over time.	Reprioritization: SMDs of importance ratings, based on the same formulas as for the then-test [49].
1.3.4 *Preference-based methods using vignettes* These methods assess the importance and value a patient explicitly places on a health state or quality-of-life dimension [3]. Patients are asked to rate (e.g., from poor to excellent) one or more anchoring vignettes, describing a particular (hypothetical) health state at different points in time [8].	Reprioritization: Statistical test of mean change in vignette ratings over time [8].	Reprioritization: SMDs of vignette ratings, based on the same formulas as for the then-test [50, 51].
2. **Latent-variable approaches**		
2.1 Structural equation models (SEMs) Latent-variable SEMs are used to test whether measurement model parameters that define the relationships between PROM indicators and their corresponding latent factors are consistent (or invariant) over time. Measurement indicators can be at the item level (where the SEM specifies the relationships between PROM items and latent factors) or subscale level (where the SEM specifies the relationships between PROM subscales and latent factors). Response shift is inferred when results indicate a lack of longitudinal measurement invariance [8, 9]. Includes: 2.1.1 *Oort’s SEM method* 2.1.2 *Schmitt’s SEM method* 2.1.3 *Other SEM method*	*Oort’s SEM method* Recalibration: This is reflected in changes in intercepts (uniform recalibration) or residual variances (non-uniform recalibration). *Explanation*: If respondents interpret the response-scale options differently at follow-up than at baseline, then domain-level mean will change even when the mean of the overall construct (i.e., the latent factor) remains constant. The corresponding intercept will change if this difference is the same for all scores of the latent factor. The domain-level residual variances will change if the difference is contingent on the latent factor score. Reprioritization: This is reflected in change in values of latent factor loadings for one or more of the domains. *Explanation*: If the importance of component domains constituting the target construct changes over time, then the relative contribution of each domain to the measurement of the overall construct (i.e., latent factor) will change. Reconceptualization: This is reflected in latent factor loadings for one or more of the domains having a value zero at one or more of the time points. *Explanation*: If respondents conceptualize the response scale differently over time, some constituting domains are absent at one time point and present at another. *Schmitt’s SEM method* Recalibration/‟beta-change”: Change in the latent factor co-variances, variances, or loadings.^c^ Reconceptualization: Changes in the pattern of latent factor loadings. Of note, change in residual variances are assumed to represent change in random error over time. *Other SEM method* Various study-specific adaptations of SEM methods to detect response shift, including principal components analysis [52–54] or exploratory and/or confirmative factor analysis methods for examining longitudinal measurement invariance [55–60].	Estimated SMDs for recalibration, reprioritization, and conceptualization response shift are based on models that adjust for a lack of longitudinal measurement invariance. SDMs were calculated in the same way for all SEM methods based on reported model parameter estimates using the formulas provided by Verdam et al. [23]).^b^ We used reported SDMs, if provided, when insufficient information was available to calculate the standardized means differences.
2.2 *Item response theory (IRT) or Rasch models* Latent factor models based on IRT or Rasch measurement theory are used to test whether measurement model parameters that define the relationships between PROM items and their corresponding latent factors are invariant over time. Response shift is inferred by a lack of invariance in discrimination power and difficulty parameters [8, 9].	Recalibration: Change in item difficulty parameters estimates or thresholds. Reprioritization (applies only to IRT): Change in item discrimination parameter estimates.	Effect sizes were not reported and could not be calculated based on information reported in the included studies
3 **Regression methods** Statistical methods that rely on regression analysis (excluding latent-variable models).		
3.1 Regression methods with classification Use of regression models to classify people as having had response shift or not.		
3.1.1 *Classification and Regression Tree (CART)* A non-parametric method that involves recursive partitioning of the longitudinal data into homogeneous subgroups (nodes) with respect to the change in the PROM scores and corresponding explanatory clinical status variables. Response shift is inferred when there is a discrepancy between clinical status and change in PROM scores or change in the relative importance of PROM domains (Sebille et al. 2021).	Recalibration: Inconsistent changes in PROM scores and clinical statu.s Reprioritization: Change in the order of importance of each domain over time.	Classification is based on the percentage of respondents identified as having had recalibration and/or reprioritization response shifts.
3.1.2 *Random forest regression* Evaluates changes in the relative contribution of PROM domains to the prediction of an outcome over time in each group. The relative importance of each domain is assessed using the average variable importance (AVI), which is the relative contribution of a domain to the prediction of an outcome in a CART averaged across several bootstrap samples. The change in the AVI for each component domain in predicting a global PROM score over time for each group is examined. Response shift is indicated by crossing curves [8].	Reprioritization: Interaction between change in AVI for different domains.	Classification is based on the percentage of respondents identified as having had reprioritization response shift.
3.1.3 *Mixed Models and Growth Mixture Models* Mixed (random effects) models are used to obtain residuals of observed minus predicted PROM scores, after which growth mixture models are used to identify latent classes of the centered residuals’ growth trajectories. Response shift is inferred when there is change in centered residuals over time [8].	General response shift effect: Discrepancy between observed and predicted scores (centered residuals having a pattern of fluctuation over time deviating from zero). Reprioritization: Effects of domain scores on global PROM scores that vary with time (i.e., interaction with time).	Classification is based on the percentage of respondents identified as having had (general or reprioritization) response shifts.
3.2 Regression methods without classification Use of regression models that do not allow for classification		
3.2.1 *Relative importance analysis* Application of logistic regression or discriminant analysis to rank PROM domains based on their relative importance in discriminating between groups. Response shift is inferred based on changes in relative importance or rank ordering of the PROM domains [8].	Reprioritization: Change in relative importance (logistic regression or discriminant analysis coefficients) of PROM domains over time.	Effect sizes were not reported and could not be calculated based on information reported in the included studies.
3.2.2 *Other regression methods without classification* A variety of study-specific applications of regression models to test for response shift effects as defined by the researchers.	Unique for each study.	Reported model R-squared (see [61] and [62] for study-specific details).
4 **Other study-specific methods** Methods that are unique to a particular study (and have not been applied in other studies). This includes various combinations of other design-based methods and other statistical methods.	Unique for each study.	Effect sizes were not reported and could not be calculated based on information reported in the included studies.

^a^The following procedures were used to standardize the mean difference, in the order of hierarchy depending on available information: (1) the standard deviation of the difference: ${\text{SD}} = {\text{SD}}_{{{\text{difference}}}} = \sqrt {{\text{SD}}_{{{\text{baseline}}}}^{2} + {\text{SD}}_{{\text{follow - up}}}^{2} - 2*r_{{{\text{baseline}},{\text{follow - up}}}} *{\text{SD}}_{{{\text{baseline}}}} *{\text{SD}}_{{\text{follow - up}}} }$, where SD = standard deviation and *r* = correlation, which was assumed to be 0.5 when the actual correlation could not be determined; (2) the pooled standard deviation: ${\text{SD}} = {\text{SD}}_{{{\text{pooled}}}} = \sqrt {{\text{SD}}_{{{\text{baseline}}}}^{2} + {\text{SD}}_{{\text{follow - up}}}^{2} - {\text{SD}}_{{{\text{baseline}}}} *{\text{SD}}_{{\text{follow - up}}} }$, where SD = standard deviation. Here, SD_pooled_ = SD_difference_ when $r_{{{\text{baseline}},{\text{follow - up}}}} = 0.5$; (3) the standard deviation of the baseline measurement: SD = ${\text{SD}}_{{{\text{baseline}}}}$; (4) when the median and interquartile range (IQR) were provided, and the mean was derived following Eq. 14 from Wan et al. (2014): $\overline{X} = \frac{{q_{1} + m + q_{3} }}{3}$, where *q*_1_ is the first quartile, *m* is the median, and *q*_3_ is the third quartile. The standard deviation was derived following Eq. 15 from Wan et al. (2014): ${\text{SD}} = \frac{{q_{3} - q_{1} }}{\eta \left( n \right)}$, where numerical values for $\eta \left( n \right)$ associated with different sample sizes are provided in Table 2 of Wan et al. (2014); (5) when confidence intervals were provided, the standard deviation was derived as follows: ${\text{SD}} = \frac{{({\text{UL}}_{{95\% {\text{CI}}}} - \overline{X})*\sqrt N }}{1.96}$, where ${\text{UL}}_{{95\% {\text{CI}}}}$ = the upper limit of the 95% confidence interval; and (6) when the *t* statistic, *t*, of a paired t test was provided, the standard deviation of the difference was derived as follows: SD$= \frac{{\sqrt N *\overline{D}}}{t}$, where $\overline{D}$ is the mean of the difference between baseline and then-test.

^b^For Schmitt’s method, recalibration, or “beta-change,” refers to a change in metric of the latent factor, which may or may not involve recalibration of any of its measurement indicators. Thus, recalibration by Schmitt’s method does not distinguish between recalibration and reprioritization as operationalized by Oort’s method.

^c^For SEMs, Recalibration SMD = $\frac{{\tau_{{{\text{post}}}} {-} \tau_{{{\text{pre}}}} }}{{{\text{SD}}}}$, where $\tau_{{{\text{post}}}}$ and $\tau_{{{\text{pre}}}}$ are the intercept or threshold values at follow-up and baseline occasions and is the standard deviation that is used to standardize the difference in intercept or threshold values. This standard deviation can be based on model parameter estimates or sample characteristics, following the same hierarchy as with the calculation of the SMD for the then-test in Eqs. 2–4. Reprioritization/reconceptualization SMD = $\frac{{\left( {\Lambda_{{{\text{post}}}} {-} \Lambda_{{{\text{pre}}}} } \right)\kappa_{{{\text{post}}}} }}{{{\text{SD}}}}$, where $\Lambda_{{{\text{post}}}}$ and $\Lambda_{{{\text{pre}}}}$ are the factor loading values at follow-up and baseline occasions, $\kappa_{{{\text{post}}}}$ is the mean of the latent variable(s) at follow-up occasion, and ${\text{SD}}$ is the standard deviation that is used to standardize the difference in factor loading values. This standard deviation was based on model parameter estimates or sample characteristics, following the same hierarchy as with the calculation of the SMD for the then-test in Eqs. 2–4.

Most studies on response shift have focused on response shift detection, and relatively fewer studies have focused on estimating the magnitudes of response shift effects. A previous scoping review by Sajobi et al. [7] on 101 studies using quantitative response shift methods published through 2016 indicated that 96 studies (95%) had detected response shift. Of these studies, 82 (85.4%) detected recalibration response shift, 20 studies (20.8%) detected reprioritization response shift, four studies (4.2%) detected reconceptualization response shift, and seven studies (7.3%) reported a general response shift effect without indicating a particular pathway. A more recent systematic review of 107 studies, also using quantitative methods, which were published between 2010 and 2020 [10] found that only 91 studies (70.5%) had detected response shift. Less than half of the studies (51 studies) overlapped with the former review by Sajobi et al. [7]. Recalibration response shift was found in 73 studies, with 27 (37%) studies using the then-test, 24 (33%) applying structural equation modeling (SEM), and 22 (30%) adopting other methods. In both reviews, reprioritization and reconceptualization response shifts were detected less frequently and if they were, they were predominantly identified by Oort’s SEM  method [11, 12].

Previous meta-analyses of response shift effects have focused on estimating the magnitudes of the effects, with results suggesting that effect sizes are relatively small on average. However, the meta-analyses also reveal substantial heterogeneity. A meta-analysis of studies published up to 2005 that used the then-test revealed Cohen’s *d* effect sizes ranging from 0.08 to 0.32 [13]. A more recent systematic review examined response shift effects in persons with cancer [14]. Seventeen of the 35 studies reported effect sizes of which 12 studies found negligible to small effect sizes, four studies found moderate effect sizes, and one study identified a single effect size of large magnitude. A systematic review on nine studies that examined response shift in people with orthopedic conditions after rehabilitation [15], found effect sizes varying in magnitude although most were small. To date, systematic reviews on the magnitudes of response shift effects included only studies focusing on either a particular response shift method (i.e., the then-test) or a specific patient population (i.e., persons with cancer or an orthopedic condition).

The above reviews reveal considerable heterogeneity in characteristics of response shift studies as they were conducted in different populations, employed different study designs, used different PROMs, and applied different response shift methods. These observations give rise to the question: What is the prevalence (i.e., relative frequency) and magnitude of response shift effects for different response shift methods and across different characteristics of response shift studies? To answer this question, it is important to consider the results from quantitative response shift studies, including results from studies for which effect sizes cannot be obtained. However, a descriptive synthesis of all quantitative response shift results has thus far not been reported.

To address this gap, we conducted a systematic review of all published quantitative studies that investigated response shift using PROMs. Our aim was to describe evidence about response shift results including distributions of response shift prevalence and, where possible, effect sizes, for different response shift methods, and population, study design, and PROM characteristics. We recognize that there continues to be a debate about the conceptualization of response shift in the QOL and health measurement literature. We therefore initiated the Response Shift – in Sync Working Group that aims to synthesize the work on response shift to date [16], including the definitional and theoretical underpinnings of response shift [17], the critical examination of response shift detection methods and their underlying operationalizations of response shift [8], and the implications of response shift for healthcare decision-making based on PROMs [6]. The descriptive systematic review reported herein is part of this initiative. With this review we do not intend to make recommendations of what response shift is or what metrics should be used. Rather, our aim is to describe and synthesize the results of response shift research to date, including the inherent heterogeneity in operationalization. This type of descriptive synthesis is important for identifying gaps, formulating new research questions, designing new longitudinal studies, and guiding future research directions.

## Methods

We conducted a systematic review (registered retrospectively in INPLASY at time of data analysis: #202290033) [18] following guidelines by Cooper, Hedge, and Valentine [19] and used the PRISMA statement as a guide for reporting the results [20].

### Search strategy and eligibility criteria

**Fig. 2:**
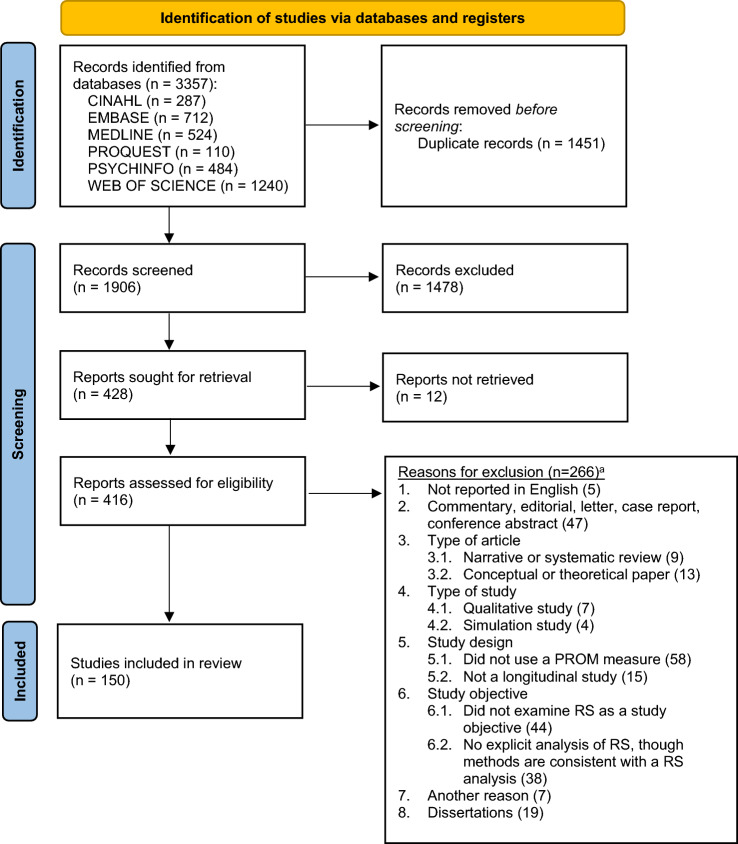
PRISMA flow diagram. *RS* response shift,* PROM* patient-reported outcome measure, *HRQOL* Health-related quality of life. ^a^Reasons are ranked by the first identified reason for exclusion. From: Page MJ, McKenzie JE, Bossuyt PM, Boutron I, Hoffmann TC, Mulrow CD, et al. The PRISMA 2020 statement: an updated guideline for reporting systematic reviews. BMJ 2021;372:n71. https://doi.org/10.1136/bmj.n71 For more information, visit: http://www.prisma-statement.org/

Studies on response shift were identified by searching the following library databases: (a) MEDLINE, PSYCINFO, and CINAHL using the EBSCO interface; (b) EMBASE using the OVID interface; (c) Social Science Citation Index using the Web of Science interface; and (d) Dissertations & Theses Global using the ProQuest interface (see Fig. 2). All searches were conducted using the same combination of the following terms and corresponding abbreviations in all indexed fields: “response shift” OR “longitudinal measurement invariance” OR “retrospective bias” OR “longitudinal differential item” OR “longitudinal DIF.” The searches were limited to English language and a date of publication before January 1, 2021. For the Social Science Citation Index, an additional limit was applied to exclude meeting abstracts. No other filters were applied to any of the searches. Duplicate records were identified and removed using the duplicate screening tool in the EPPI Reviewer Platform [21]. Manuscripts that reported on errata or on the same study as reported in another manuscript were identified as duplicates during the data extraction process after confirming that no additional relevant information could be extracted. We retained the manuscripts that reported the most detailed results.

We aimed to include all longitudinal quantitative studies that examined response shift using a PROM. Exclusion criteria were sequentially applied in the order shown in Fig. 2. The titles and abstracts of each citation were randomly assigned for independent screening by two team members (RS, LR, MEGV, VS, MAGS), all of whom were thoroughly familiar with response shift, using the EPPI Reviewer platform [21]. The full text was subsequently retrieved for each citation identified as potentially relevant and each was screened randomly by two of the same team members. Disagreements were reconciled via consensus.

### Data extraction

Data extraction for each included study was completed by one of three team members (LR, MV, RS). Ambiguities were discussed among team members to achieve agreement. Study-level information was extracted using the EPPI reviewer application and detailed information about each response shift effect was extracted and entered into a spreadsheet. The following study-level data extraction characteristics (see Tables 2, 3, 4, 5, 6 for details) were defined in advance and further refined to resolve emerging ambiguities during the data extraction process:

1. Response shift methods: design-based methods, latent-variable methods, regression methods, and study-specific methods (see Table 1 for details).
2. Population characteristics: sex, age, medical condition, and intervention.
3. Study design characteristics: experimental/observational, primary/secondary analysis, sample size, and duration of time between measurement occasions.
4. PROMs characteristics (only including PROMs used for the response shift analysis): name of PROM, type of PROM (generic/disease-specific/individualized/other, where the category individualized PROMs supersedes the categories generic/disease-specific PROMs), and PROM domains.
5. Study results: detection (yes/no) and magnitude (see under statistical analyses) of recalibration, reconceptualization, and reprioritization and dependencies, i.e., whether the response shift effect pertained to a subsample (or group) of an overall sample reported in the same manuscript or the same or overlapping sample from another study.

**Table 2:** Prevalence of response shift results by method

	Study-level results		Effect-level results					
	Total effects		Total effects		Recalibration		Reprioritization and/or Reconceptualization, or Unknown^a^	
	*N*	% RS detected	*N*	% RS detected	*N*	% RS detected	*N*	% RS detected
**Design-based methods**								
Then-test	82	86.6	1004	39.2	1004	39.2	n/a	n/a
Individualized methods	12	100	31	74.2	9	44.4	22	86.4
Other methods	11	72.7	214	10.7	12	50.0	202	8.4
**Latent-variable models**								
SEM	44	79.5	3139	7.7	986	16.4	2153	3.7
IRT/Rasch	3	100	81	25.9	61	13.1	20	65.0
**Regression methods**								
With classification	11	81.8	44	81.8	8	100.0	36	77.8
Without classification	13	76.9	351	14.5	5	40.0	346	14.2
**Other study-specific methods**	4	50.0	4	50.0	n/a	n/a	4	50.0
**Total**	150	86.7	4868	16.3	2085	28.0	2783	7.5

^a^*Unknown* includes several effects for which the pathway was unknown due to it not being explicitly reported. *N* = the number of studies or response shift effects. For each study, response shift methods were only counted when results about the response shift effects were reported. For studies that reported the same results in multiple manuscripts, only the results of the first published study were counted. % RS detected = the percentage of detected response shift effects of # studies or # response shift effects that were investigated or possible. SEM = Structural Equation Model. IRT/Rasch = Item Response Theory/Rasch Measurement Model. n/a = not applicable.

**Table 3:** Prevalence and magnitude of effect sizes by method

RS metric and method	Study-level results		Effect-level results							
	Total effects		Total effects		Recalibration effects			Reprioritization and/or Reconceptualization effects, or unknown^a^		
	Prevalence		Prevalence		Prevalence		Magnitude	Prevalence		Magnitude
	*N*	% RS detected	*N*	% RS detected	*N*	% RS detected	ES	*N*	% RS detected	ES
**Cohen’s *d***^b^	**91**	**89.0**	**1062**	**46.1**			**Median (IQR)**			**Median (IQR)**
Design-based methods: then-test	72	87.5	929	40.4	929	40.4	0.22 (0.10–0.38)	n/a	n/a	n/a
Design-based methods: individualized^c^	1	100.0	7	28.6	7	28.6	0.03 (0.03–0.11)	n/a	n/a	n/a
Design-based methods: other	3	66.7	15	46.7	4	50.0	0.28 (0.09–0.45)	11	45.5	0.09 (0.05–0.17)
Latent-variable models: SEM	19	100.0	111	95.5	84	98.8	0.22 (0.14–0.35)	27	85.2	0.10 (0.10–0.14)
**R-squared: median** **(min–max)**	**2**	**100.0**	**27**	**25.9**			**median (IQR)**			**median (min–max)**
Regression without classification	2	100.0	27	25.9	n/a	n/a	n/a	27	25.9	0.01 (0.00–0.02)
**Classification: % respondents with RS**	**17**	**100.0**	**27**	**100.0**			**% sample with RS**			**% sample with RS**
Design-based methods: individualized	8	100.0	13	100.0	n/a	n/a	n/a	13	100.0	68.2
Design-based methods: other	1	100.0	1	100.0	n/a	n/a	n/a	1	100.0	48.6
Regression with classification	6	100.0	11	100.0	2	100.0	30.3	9	100.0	15.2
Other study-specific methods	2	100.0	2	100.0	n/a	n/a	n/a	2	100.0	13.3
**Other effect size metric**	**3**	**66.7**	**14**	**92.9**						
Design-based methods: Individualized	1	100.0	1	100.0	1	100.0	n/a	0	n/a	n/a
Latent-variable models: SEM	1	100.0	12	100.0	7	100.0	n/a	5	100.0	n/a
Other study-specific methods	1	0.0	1	0.0	n/a	n/a	n/a	1	0.0	n/a

Based on a total of 105 studies and 1130 effects for which effect sizes could be determined. *N* = the corresponding # of studies or response shift effects (which are the same for prevalence and magnitudes of the effects). The # of studies (first column) do not add up to 105 or to their respective subtotals (bolded rows) because some studies applied more than one response shift method. % RS detected = the percentage of detected response shift effects of # studies or # response shift effects. % sample = percentage of the pooled sample size (total number of people across studies). SEM = Structural Equation Model.

n/a = not applicable because either the response shift pathway could not be investigated due to the method used or the results could not be discerned from what was reported in the manuscript. IQR = interquartile range.

^a^*Unknown* includes several effects for which the pathway was unknown due to it not being explicitly reported.

^b^Cohen’s*d* was calculated for 951 of the 1062 effects based on the statistical information reported following the procedures described in Table 1. The remaining 111 effects could not be calculated due to inadequate statistical information, in which case we relied on the Cohen’s d effect sizes reported in the manuscript (of these 89 effect sizes were explicitly reported as standardized mean differences and 22 effect sizes were assumed to be standardize mean differences based on overall description of the methods).

^c^Based on one study where the then-test method was applied to calculate SMDs based on the SEIQoL [34].

**Table 4:** Prevalence and magnitude of response shift results across population characteristics

Population characteristics	Study-level results		Effect-level results									
	Total effects		Total effects		Recalibration effects				Reprioritization and/or Reconceptualization effects, or unknown^a^			
	Prevalence^b^		Prevalence^b^		Prevalence^b^		Magnitude^c^		Prevalence^b^		Magnitude^c^	
	*N*	% RS detected	*N*	% RS detected	*N*	% RS detected	*N*	Median ES (IQR)	*N*	% RS detected	*N*	Median ES (IQR)
Sex												
Mixed	121	85.1	3734	14.5	1428	26.1	522	0.23 (0.10–0.43)	2306	7.2	30	0.09 (0.01–0.17)
Only female	13	84.6	724	23.3	473	32.1	381	0.17 (0.09–0.28)	251	6.8	n/a	n/a
Only male	12	66.7	206	25.7	135	34.1	101	0.31 (0.22–0.42)	71	9.9	6	0.12 (0.05–0.25)
Other/unknown	8	100.0	204	14.7	49	26.5	20	0.36 (0.19–0.57)	155	11.0	2	0.12 (0.10–0.14)
Age												
Mostly adults	100	84.0	3172	17.7	1410	27.6	754	0.21 (0.10–0.36)	1762	9.7	17	0.14 (0.10–0.22)
Mostly older adults	34	88.2	825	16.6	342	30.7	184	0.24 (0.10–0.44)	483	6.6	16	0.05 (0.02–0.12)
Mostly children/adolescents	8	87.5	227	12.3	93	30.1	28	0.04 (0.00–0.14)	134	0.0	n/a	n/a
Other/unknown	11	90.9	644	10.4	240	25.8	58	0.28 (0.20–0.53)	404	1.2	5	0.03 (0.01–0.06)
Medical condition												
No	9	88.9	201	12.4	47	38.3	25	0.23 (0.12–0.41)	154	4.5	7	0.05 (0.04–0.09)
Yes: cancer	45	93.3	1961	18.6	1054	30.3	665	0.22 (0.10–0.35)	907	5.1	14	0.08 (0.01–0.14)
Yes: orthopedic	9	77.8	78	52.6	77	51.9	77	0.43 (0.23–0.82)	1	100.0	n/a	n/a
Yes: stroke	11	90.9	520	9.2	152	13.2	21	0.28 (0.18–0.55)	368	7.6	4	0.08 (0.05–0.12)
Yes: mental health	9	77.8	472	5.9	159	10.7	1	0.49 (0.49–0.49)	313	3.5	n/a	n/a
Yes: other	67	83.6	1636	17.4	596	28.5	235	0.18 (0.09–0.31)	1040	11.1	13	0.17 (0.08–0.25)
Intervention												
No/unclear	48	83.3	1837	10.6	605	18.5	217	0.21 (0.10–0.37)	1232	6.7	13	0.09 (0.05–0.14)
Yes: medical	69	88.4	2315	21.0	1176	33.3	689	0.22 (0.11–0.38)	1139	8.3	17	0.03 (0.00–0.11)
Yes: psychological	20	90.0	567	10.9	222	19.8	65	0.23 (0.11–0.41)	345	5.2	n/a	n/a
Yes: other/unspecified	13	84.6	149	32.9	82	43.9	53	0.16 (0.09–0.31)	67	19.4	8	0.20 (0.10–0.39)

^a^*Unknown* includes several effects for which the pathway was unknown due to it not being explicitly reported.

^b^*Prevalence* is based on a total of 150 studies and 4868 effects that were investigated or possible. *N* = # of studies or response shift effects. The # of studies may not add up to 150 (first column) because some studies implemented multiple methods or had multiple samples that were counted separately. % RS detected = the percentage of detected response shift effects of # studies conducted or # response shift effects that were investigated or possible.

^c^*Magnitude* is based on 91 studies and 1062 effects for which Cohen’s *d* could be determined. *N* = the # of these response shift effects. ES = Cohen’s *d.* IQR = interquartile range. n/a = not applicable because either the response shift pathway could not be investigated due to the method used or the results could not be discerned from what was reported in the manuscript.

*Example*: For “mixed sex,” there are a total of 121 studies of which 85.1% had detected response shift and 3734 effects of which 14.5% had detected response shift. There were 1428 recalibration effects, of which 26.1% had detected response shift and 522 for which a Cohen’s *d* was obtained. There were also 2306 reprioritization and/or reconceptualization or unknown effects, of which 7.2% had detected response shift and 30 for which a Cohen’s *d* could be determined.

**Table 5:** Prevalence and magnitude of response shift results for different study design characteristics

Study design characteristics	Study-level results		Effect-level results									
			Total effects		Recalibration effects				Reprioritization and/or Reconceptualization effects, or unknown^a^			
	Prevalence^b^		Prevalence^b^		Prevalence^b^		Magnitude^c^		Prevalence^b^		Magnitude^c^	
	*N*	% RS detected	*N*	% RS detected	*N*	% RS detected	*N*	Median ES (IQR)	*N*	% RS detected	*N*	Median ES (IQR)
Design												
Observational	122	88.5	3978	16.2	1724	28.1	879	0.21 (0.10–0.36)	2254	7.1	33	0.10 (0.04–0.17)
Experimental	28	78.6	890	16.5	361	27.7	145	0.26 (0.16–0.41)	529	8.9	5	0.03 (0.01–0.06)
Data analysis												
Primary analysis	90	87.8	1590	30.2	1058	39.7	872	0.22 (0.11–0.38)	532	11.3	15	0.14 (0.08–0.25)
Secondary analysis	59	84.7	3268	9.5	1027	16.0	152	0.19 (0.09–0.32)	2241	6.6	23	0.05 (0.02–0.10)
Unknown	1	100.0	10	10.0	n/a	n/a	n/a	n/a	10	10.0	n/a	n/a
Sample sizes (Binned)												
Q1 (< 57)	59	76.3	1121	16.1	584	23.3	391	0.26 (0.13–0.45)	537	8.4	5	0.03 (0.01–0.06)
Q2 (57–254)	79	82.3	1353	23.4	668	35.5	393	0.22 (0.10–0.38)	685	11.5	13	0.14 (0.10–0.20)
Q3 (255–410)	34	85.3	1262	15.8	472	32.0	194	0.15 (0.09–0.24)	790	6.2	8	0.10 (0.07–0.35)
Q4 (> 411)	27	77.8	1132	8.4	361	16.6	46	0.20 (0.12–0.35)	771	4.5	12	0.05 (0.02–0.13)
Time period classification												
< 1 month	15	80.0	488	13.1	205	24.9	95	0.23 (0.09–0.32)	283	4.6	7	0.01 (0.00–0.03)
1–6 months	23	91.3	427	19.9	110	43.6	49	0.40 (0.22–0.71)	317	11.7	11	0.05 (0.04–0.10)
> 6–12 months	36	72.2	905	12.3	347	21.0	78	0.18 (0.09–0.39)	558	6.8	n/a	n/a
> 12 months	90	87.8	2604	18.2	1211	29.7	733	0.22 (0.11–0.38)	1393	8.1	16	0.13 (0.09–0.28)
Not reported	12	83.3	444	13.3	212	24.5	69	0.16 (0.10–0.28)	232	3.0	4	0.18 (0.13–0.24)

^a^*Unknown* includes several effects for which the pathway was unknown due to it not being explicitly reported.

^b^*Prevalence* is based on a total of 150 studies and 4868 effects that were investigated or possible. *N* = # of studies or response shift effects. The # of studies may not add up to 150 (first column) because some studies implemented multiple methods or had multiple samples that were counted separately. % RS detected = the percentage of detected response shift effects of # studies conducted or # response shift effects that were investigated or possible.

^c^*Magnitude* is based on 91 studies and 1062 effects for which Cohen’s *d* could be determined. *N* = the # of these response shift effects. ES = Cohen’s *d.* IQR = interquartile range. n/a = not applicable because either the response shift pathway could not be investigated due to the method used or the results could not be discerned from what was reported in the manuscript.

*Example:* For observational study designs, there are a total of 122 studies of which 88.5% had detected response shift and 3978 effects of which 16.2% had detected response shift. There were 1724 recalibration effects, of which 28.1% had detected response shift and 879 for which a Cohen’s *d* was obtained. There were also 2254 reprioritization and/or reconceptualization or unknown effects, of which 7.1% had detected response shift and 33 for which a Cohen’s *d* was obtained.

**Table 6:** Prevalence and magnitude of response shift results for different PROM characteristics

PROM characteristics	Study-level results		Effect-level results										
			Total effects		Recalibration effects				Reprioritization and/or Reconceptualization effects, or Unknown^a^				
	Prevalence^b^		Prevalence^b^		Prevalence^b^		Magnitude^c^		Prevalence^b^			Magnitude^c^	
	*N*	% RS detected	*N*	% RS detected	*N*	% RS detected	*N*	ES (IQR)	*N*		% RS detected	*N*	ES (IQR)
PROM types													
Generic PROMs^d^	**76**	**84.2**	**1971**	**14.7**	**769**	**23.0**	**324**	**0.23 (0.10–0.41)**	**1202**		**9.3**	**18**	**0.10 (0.01–0.14)**
#1 SF family^e^	47	83.0	1248	16.3	428	25.2	182	0.23 (0.11–0.44)	820		11.6	18	0.10 (0.01–0.14)
#2 EQ 5D^e^	14	78.6	112	19.6	76	18.4	64	0.20 (0.08–0.32)	36		22.2	n/a	n/a
#3 Other	24	75.0	611	10.5	265	20.8	78	0.26 (0.11–0.41)		346	2.6	n/a	n/a
Disease-specific PROMs^d^	**57**	**80.7**	**1431**	**20.8**	**755**	**33.8**	**457**	**0.19 (0.10–0.33)**		**676**	**6.4**	**4**	**0.17 (0.09–0.47)**
#1 EORTC family^e^	17	88.2	616	25.2	404	35.4	315	0.17 (0.09–0.28)		212	5.7	4	0.17 (0.09–0.47)
#2 Oral impact profile^e^	4	75.0	83	33.7	48	43.8	27	0.21 (0.17–0.29)		35	20.0	n/a	n/a
#3 Other	37	78.4	732	15.7	303	30.0	115	0.29 (0.12–0.48)		429	5.6	n/a	n/a
Individualized PROM	10	90.0	30	86.7	8	87.5	6	0.23 (0.20–0.30)		22	86.4	n/a	n/a
Other type of PROM	46	69.6	1436	12.5	553	26.2	275	0.25 (0.13–0.37)		883	3.9	16	0.07 (0.03–0.14)
PROM domains													
General health/QOL	95	68.4	561	28.0	295	32.2	182	0.23 (0.09–0.41)		266	23.3	3	0.14 (0.14–0.20)
Physical	96	70.8	1823	15.5	799	27.5	429	0.20 (0.10–0.35)		1024	6.1	16	0.08 (0.02–0.28)
Psychological: depression	8	62.5	153	11.1	58	27.6	12	0.27 (0.21–0.33)		95	1.1	n/a	n/a
Psychological: other	85	62.4	1121	13.5	440	25.0	199	0.23 (0.11–0.33)		681	6.0	12	0.09 (0.03–0.13)
Social	57	59.6	534	13.7	203	27.1	78	0.23 (0.13–0.35)		331	5.4	5	0.10 (0.05–0.11)
Pain	48	64.6	236	28.0	104	55.8	76	0.33 (0.16–0.54)		132	6.1	n/a	n/a
Other	30	56.7	440	10.5	186	16.1	86	0.15 (0.07–0.27)		254	6.3	2	0.05 (0.00–0.09)

^a^*Unknown* includes several effects for which the pathway was unknown due to it not being explicitly reported.

^b^*Prevalence* is based on a total of 150 studies and 4868 effects that were investigated or possible. N = # of studies or response shift effects. The # of studies may not add up to 150 (first column) because some studies implemented multiple methods or had multiple samples that were counted separately. % RS detected = the percentage of detected response shift effects of # studies conducted or # response shift effects that were investigated or possible.

^c^*Magnitude* is based on 91 studies and 1062 effects for which Cohen’s *d* could be determined. *N* = the # of these response shift effects. ES = Cohen’s *d.* IQR = interquartile range. n/a = not applicable because either the response shift pathway could not be investigated due to the method used or the results could not be discerned from what was reported in the manuscript.

^d^Bolded values indicate pooled results for all generic or disease-specific PROMs. SF = Short Form Health Survey, EQ-5D = EuroQol 5 Dimensions, EORTC = European Organization for Research and Treatment of Cancer.

^e^First and second most frequent PROM based on the # of studies in which the PROM was used (the # of effects were used when two PROMs are tied based on the # of studies alone).

*Example:* For “Generic PROMs,” there are a total of 76 studies of which 84.2% had detected response shift and 1971 effects of which 14.7% had detected response shift. There were 769 recalibration effects, of which 23.0% had detected response shift and 324 for which a Cohen’s *d* was obtained. There were also 1202 reprioritization and/or reconceptualization or unknown effects, of which 9.3% had detected response shift and 18 for which a Cohen’s *d* was obtained.

### Statistical analyses

In all studies, authors concluded whether response shift was found or not, although the conclusions may have been based on different grounds, e.g., statistical significance, where different studies adopted different alpha levels or verbal conclusions in the absence of statistical tests. We followed the authors’ conclusions regarding the existence or non-existence of a response shift effect. Where possible, we determined the magnitude of each response shift effect based on reported statistical information from which an effect size could be derived. Table 1 includes a description of response shift detection and effect size calculation (if possible) for each method. We used reported effect sizes, if provided, when insufficient information was available to calculate effect sizes. Standardized mean differences (Cohen’s *d*) were calculated for the then-test and latent-variable methods based on information reported in each study based on the difference between baseline (${\overline{X} }_{1})$ and follow-up (then-test) (${\overline{X} }_{2})$ scores as follows: Cohen’s *d* = $\frac{{\overline{X} }_{1}-{\overline{\overline{X}}}_{2}}{\text{SD}}$ (where SD = standard deviation). For some studies, this meant that we first had to transform medians, interquartile ranges (IQR), and *t* or *z* statistics into means and standard deviations [19, 22]. We used the following hierarchy to standardize the mean difference, based on (1) the standard deviation of the difference, (2) the pooled standard deviation, or (3) the standard deviation of the baseline measurements (see footnote to Table 1 for details). For SEM, response shift effects were based on parameter estimates of models that adjust for a lack of longitudinal measurement invariance (for more information see [23]). All effect sizes were converted to absolute values. We followed Cohen’s guidelines [24] to interpret effect sizes of 0.2, 0.5, and 0.8 to be small, moderate, and large, respectively. For regression methods that do not use classification of people as having response shift or not, the reported R-squared was used as a measure of effect size, with values of 0.01, 0.06, and 0.14 being indicative of a small, moderate, and large magnitude, respectively [24]. For regression-based response shift methods that do use classification, the proportion of people having undergone response shift was extracted as an indication of the magnitude of effects.

Response shift results and effect sizes were summarized for recalibration and reprioritization or reconceptualization effects at different levels of analysis (study and effect levels). Accordingly, the synthesis focused on describing distributions of prevalence (relative frequency) and magnitude of response shift effects, based on (a) the proportion of studies detecting response shift (study level) and (b) the proportion of response shift effects identified (effect level) for different response shift methods and population, study design, and PROM characteristics. Consistent with our descriptive aim and recognizing the inherent heterogeneity in operationalizations, we used non-parametric statistics to describe the distributions of the effect sizes, including their medians and IQRs for continuous effect sizes and percentages for classification (i.e., we did not pool effect sizes statistically).

Although we sought to describe all response shift effects, we also wanted to account for situations where multiple analyses and studies were done on the same sample. To do so, we first conducted the analyses based on all response shift effects and subsequently repeated the same analyses on the subset of response shift effects from unrelated studies and samples that do not overlap with samples from the same (e.g., subsamples) or other studies (with details reported in Supplementary Tables S1 to S5). Studies were considered related when analyses from different studies are conducted on the same or overlapping samples or when the same results are reported in multiple manuscripts. For related studies, only the first (original) study was counted. Independent samples do not have overlap with other samples. When samples are overlapping, only the overall sample was counted (subsamples were not counted). Analyses were conducted using SPSS [25] with violin plots created using the ggplot2 package in R [26].

## Results

### Studies

Of the 1906 records screened, 150 studies fulfilled the eligibility criteria and were included (see Fig. 2). Of these studies, 125 were unrelated to any of the other studies and 25 related studies involved analyses of the same or overlapping samples, of which 9 were identified as the primary (first published) studies and 16 as secondary (related studies that were published after the corresponding primary study). We identified a total of 4868 response shift effects (Table 2), of which 917 were from secondary related studies and 284 from primary related studies (3667 effects were from unrelated studies). Results of the 150 studies and 4868 response shift effects are described first, followed by a description of the results based on the 134 unrelated and primary related studies and 3579 response shift effects from independent samples (excluding 372 effects from subsamples), with further details provided in Supplementary Tables S1 to S5.

### Response shift methods

#### Prevalence

Of 150 studies, 130 (86.7%) reported detection of one or more response shift effects (Table 2), based on criteria defined by the authors. However, response shift effects were detected for only 793 (16.3%) of the total 4868 effects investigated. Most response shift results were based on 82 studies that utilized the then-test method, with 86.6% of the studies and 39.2% of 1004 corresponding effects resulting in detection of recalibration response shift. SEM methods were applied in 44 of the studies of which 79.5% resulted in detection of at least one response shift effect. However, only 7.7% of all corresponding 3139 effects revealed response shift, including 16.4% of 986 recalibration effects and 3.7% of 2153 reprioritization or reconceptualization effects.

Other methods were less frequently applied, ranging from 3 to 13 studies. When considering methods that were based on at least 10 studies (i.e., not IRT/Rasch and other study-specific methods), the highest percentage of detected response shift effects at study level was found for individualized methods (100%, 12 studies). At effect level, the percentage of detected response shift effects was also relatively high for individualized methods (74.2%) despite the small number of 31 effects. In general, the prevalence of response shift detection was lower when the number of investigated response shift effects was larger for all response shift pathways.

#### Magnitude

**Fig. 3:**
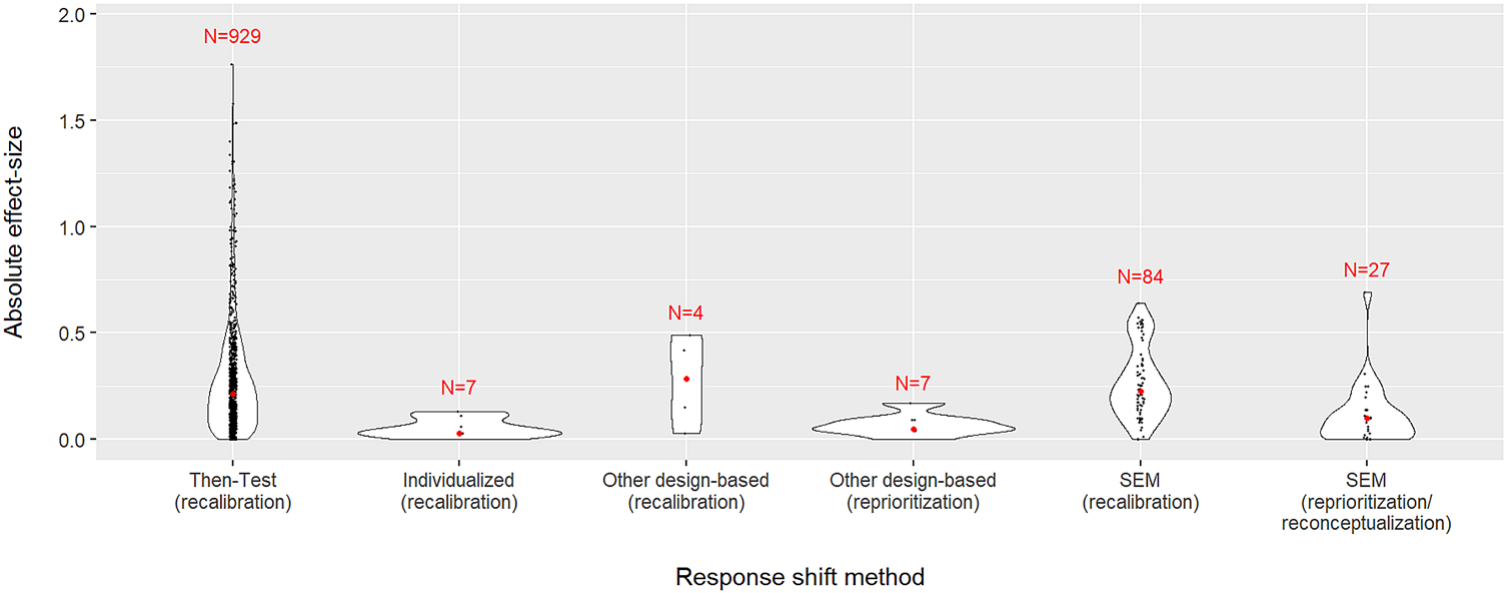
Distribution of absolute effect sizes across types of response shift methods. *Note* All violin plots have the same area, which is determined by the distribution of effects within each method. Four effect sizes for which the response shift pathway is unknown are excluded. Two extreme effect sizes of 6.9 [63] and 2.9 [64] for the then-test are not shown to ensure other distributions remain discernable (instead of becoming flat lines). The outliers were only removed for this visualization, they were included in the statistical analysis

Effect sizes could be determined for 105 (70.0%) of the studies and a total of 1130 response shift effects, with 96 (91.4%) of these studies resulting in detection of 537 (47.5%) response shift effects. Cohen’s *d* (standardized mean difference) was the most common effect size metric, which was obtained for 1062 effects from 91 studies (see Table 3). Most of these effect sizes were based on studies using the then-test (72 studies and 929 effects), resulting in an overall median effect size of 0.22 with substantial dispersion (IQR 0.10–0.38) for recalibration effects. Cohen’s *d* effect sizes were also determined for 111 effects from 19 studies using SEM, where response shift was detected for all of these studies and 95.5% of the effects, with median effect sizes of 0.22 (IQR 0.14–0.35) for recalibration and 0.10 (IQR 0.00–0.14) for reprioritization or reconceptualization. Other methods enabling the calculation of Cohen’s *d* effect sizes included other design-based methods (3 studies and 15 effects) and individualized methods (1 study and 7 effects) (see Table 3). The distribution of effect sizes across these methods is visualized as violin plots in Fig. 3. Additionally, two studies (27 effects) provided the R-squared statistic as an effect size for regression methods without classification, resulting in a median effect size of 0.01 (IQR 0.00–0.02) for reprioritization/reconceptualization. Response shift effect sizes could also be obtained for 27 effects from 17 studies using classification methods, of which all resulted in detection of response shifts. The greatest effect size was obtained for the design-based individualized classification method, which also had the largest number of effects (13), resulting in 68.2% of the pooled sample size indicating reprioritization/reconceptualization response shift. Finally, three studies (14 effects) reported study-specific effect size metrics (see Table 3).

### Study population characteristics

#### Prevalence

At study level, most studies involved participants with mixed sex (121 studies), who were mostly adults (100 studies), with a medical condition (141 studies), and/or undergoing a medical intervention (69 studies) (Table 4). The study-level prevalence of detected response shift ranged from 66.7% (only male; 12 studies) to 90.0% (medical condition: stroke; 11 studies), when excluding population characteristics with fewer than 10 studies. The corresponding effect-level prevalence values were much lower and ranged from 5.9% (mental health condition; 472 effects) to 32.9% (other/unspecified intervention; 149 effects) for population characteristics with minimally 100 effects.

#### Magnitude

When considering population characteristics for which at least 100 effects sizes could be determined, median recalibration effect sizes ranged from 0.17 (IQR 0.09–0.28) (381 effects for female samples) to 0.31 (IQR 0.22–0.42) (101 effects for male samples). The reprioritization or reconceptualization effect sizes are based on fewer effects (ranging from 2 to 30 per population characteristic), with median effect sizes ranging from 0.03 (IQR 0.00–0.11), based on 17 effects from samples with medical interventions, to 0.20 (IQR 0.10–0.39) for 8 effects from samples with other/unspecified interventions.

### Study design characteristics

#### Prevalence

Most studies employed an observational design (122 studies), conducted primary analysis (90 studies), had a sample size between 57 and 254 (79 studies), and employed an observation period greater than 12 months (90 studies). Across the four study design characteristics, the study-level prevalence of detected response shift ranged from 72.2% (time period from 6 to 12 months) to 91.3% (time period from 1 to 6 months) (excluding the one study with unknown data analysis) (Table 5). Again, the corresponding effect-level prevalence values were lower and ranged from 8.4% (1132 effects for sample size > 411) to 30.2% (1590 effects for primary analysis).

#### Magnitude

When considering study design characteristics with at least 100 effect sizes, the smallest median recalibration effects (Cohen’s *d* effect sizes) was 0.15 (IQR 0.09–0.24) based on 194 effects from studies with sample sizes between 255 and 410. The largest median effect size was 0.26 (IQR 0.16–0.41) based on 145 effects from studies adopting an experimental design and 0.26 (IQR 0.13–0.45) based on 391 effects for studies with sample sizes less than 57. The reprioritization or reconceptualization effect sizes are based on fewer effects (ranging from 4 to 33 per study design characteristic), with median effect sizes ranging from 0.01 (for 7 effects from studies employing a time frame of < 1 month) to 0.14 (based on 15 effects from studies conducting primary analysis and 13 effects of studies using a sample size between 57 and 254) (excluding the time period classification “not reported,” since this is essentially a missing data category).

### PROM characteristics

#### Prevalence

With respect to PROM type, most studies employed a generic PROM (76 studies) with the SF family of PROMs (47 studies) and the EQ-5D (14 studies) being most prevalent (Table 6). Disease-specific PROMs were used in 57 studies, with the EORTC measures being used most often (17 studies). Of the PROM domains, physical (96 studies), general health/QOL (95 studies), and psychological other than depression (85 studies) domains were measured most frequently. When excluding PROM types used in 10 or less studies, study-level prevalence of detected response shift for PROM types ranged from 69.6% (46 studies using other types of PROMs) to 90.0% (10 studies using individualized PROMs). Regarding the different PROM domains, study-level prevalence was within a range of 12 percentage points for the major domains, ranging from 56.7% for ‘other’ domain to 70.8% for the physical domain (excluding those with 10 or less studies).

When considering PROM types with at least 100 effect sizes, the effect-level prevalence of detected response shift for PROM types ranged from 10.5 (611 effects for ‘other’ generic PROMS) to 25.2% (616 effects generated by studies employing EORTC measures). The corresponding effect-level prevalence values for the PROM domains (excluding those with less than 100 effects) ranged from 10.5% for other PROM domains (440 effects) to 28% for both general health/QOL and pain (561 and 236 effects, respectively).

#### Magnitude

When considering PROM types with at least 100 effect sizes, median recalibration effect sizes (Cohen’s *d*) ranged from 0.17 (IQR 0.09–0.28) for 315 effects from studies using the EORTC PROMs to 0.29 (IQR 0.12–0.48) for 115 effects based on studies using ‘other’ disease-specific PROMs. Median reprioritization/reconceptualization effect sizes ranged from 0.07 (IQR 0.03–0.14) based on 16 effects for other types of PROMs to 0.17 (IQR 0.09–0.47) based on 4 effects for the EORTC family. For PROM domains, median recalibration effects based on at least 100 effects, ranged from 0.20 (IQR 0.10–0.35) based on 429 effects for the physical domain to 0.23 (IQR 0.09–0.41) based on 182 effects for general health/QOL and 0.23 (IQR 0.11–0.33) for 199 effects for the psychological domain other than depression. The reprioritization or reconceptualization effect sizes are based on fewer effects (ranging from 2 (‘other’ PROM domain) to 18 (SF family) across all PROM characteristics), with median effect sizes ranging from 0.05 (IQR 0.00–0.09) based on 2 effects for the PROM domain other to 0.17 (IQR 0.19 – 0.47) based on 4 effects for the EORTC PROMs.

### Unrelated studies and non-overlapping samples

Prevalence and effect size estimates were similar for most methods and population, study design, and PROM characteristics when only unrelated studies and non-overlapping samples were considered (see Table S1–S5). Study-level differences in prevalence ranged from 0.0 to 5.9% (when considering methods and characteristics with at least 10 studies) for most methods and characteristics, with the exception of the characteristics ‘only males’ or a ‘sample size of 255–410,’ which had greater % prevalence when considering only independent effects. Effect-level differences in prevalence ranged from 0.0 to 14.9%, with 15 of the effect-level differences exceeding 5% (when considering methods and characteristics with at least 100 effects). The median difference in Cohen’s *d* estimates of recalibration response shift is 0.02 (when considering methods and characteristics with at least 100 effects), with the largest differences for effects for the characteristics ‘unknown sex’ and a ‘mental health condition.’ For reprioritization/recalibration, the number of effects was too small to warrant meaningful comparisons.

## Discussion

To further find the field of response shift research, this study described variation in prevalence of response shift results and where possible, magnitude of response shift effects for quantitative studies using PROM data. Consistent with earlier reviews [7, 10], the most frequently applied response shift method was the then-test, followed by SEM, other design-based methods, regression methods without classification, individualized methods, regression methods with classification, IRT/Rasch, and other study-specific methods. Most studies reported detection of one or more response shift effects. However, response shift effects were detected for only a sixth of all effects investigated. Clearly study-level prevalence is expected to be higher than effect-level prevalence because a study was classified as having detected response shift when response shift was detected for any of the multiple effects being studied. However, it is noteworthy that the prevalence distributions at the study level as compared to the effect-level were also different across the different methods, population, study design, and PROM characteristics, i.e., different methods and characteristics would be identified as having higher or lower prevalence of response shift effects when this is determined at the study as compared to the effect-level. Individual studies and previous reviews primarily focused on study-level results, drawing binary conclusions about whether response shift is present or not. Our results show that such a one-sided focus may be misleading, and only the combined information at study and effect level provides a comprehensive overview of response shift results.

Effect sizes were determined for 105 of the 150 studies. Whereas, the median effect sizes varied per method, population, study design, and PROM characteristic, they were all of a small magnitude, with most recalibration effect sizes between 0.20 and 0.30 and reprioritization/reconceptualization effect sizes rarely exceeding 0.15. There may be methodological explanations for the small effect sizes found. One explanation would be the presence of heterogeneous samples where response shifts may occur at the individual level but in different directions that cancel each other out at the group level. Nonetheless, given the small median effect sizes, we need to acknowledge that there are empirical situations where the impact of response shift may be quite small, or even negligible, when we are only interested in results for the entire group. But even in such contexts, there is reason to consider such effect sizes as relevant. Many effects in PROM research have a comparable magnitude. For example, a systematic review aimed to investigate, among other things, whether patients who share their responses to PROMs with their health care provider have better health. The results indicated small effect sizes for such PROMs feedback on patient-reported health outcomes [27]. This review illustrates that the target signal (in this case PROMs feedback) may not be substantially different in magnitude than other processes triggered using PROMs, such as response shift.

The most striking finding is that the effect sizes varied widely, ranging from zero to large, both within and between studies. This observation draws attention to the importance of considering the dispersion of response shift effects within studies rather than relying exclusively on within-study pooled results. The large variability in effect size estimates may also have methodological explanations, including variability in response shift methods (e.g., SEM methods lead to smaller effect sizes than then-tests), study populations, sample sizes, and PROMs. The proportion of variability in effect sizes attributable to such study characteristics is currently unknown. When part of the variability in effect sizes would indeed be caused by differences between study populations, then substantial effects may be experienced by some groups or individuals or in specific contexts or with certain PROMs. Ignoring such variability would not pay credit to the experiences of the respondents and the richness of the data. In other words, variability can be highly meaningful. A parallel can be drawn with precision medicine, e.g., in cancer treatment. Whereas some treatments may hardly affect the survival of a particular population, further investigation into the variability of effects may reveal that the treatment can be highly effective in a subgroup of patients whose tumor DNA matches the working mechanisms of those treatments. Hence, information about the variability of treatment effects enables the development of targeted therapy, which ultimately results in more life years gained. Whereas research into response shift will not lead to such dramatic gains, we may need to be moving into ‘precision methodology’ for response shift. The key message is that, rather than focusing on effect sizes for the entire group, we should focus on describing and understanding variability in effects: in terms of identification (who experience response shift?), magnitude (to what extent?), and under which circumstances. Moreover, arguments around social justice and societal inequalities would require such subgroup analyses investigating whether response shift effects systematically favor or disadvantage some groups of people [6, 28].

A number of limitations of this systematic review merit attention. We omitted studies that were not reported in English. We also cannot preclude the possibility that we may have missed relevant papers despite our extensive literature search. The synthesis of included studies was challenged due to different operationalizations of response shift and inadequate reporting of study results and/or methodology. A substantial number of studies required a disproportionate amount of effort from the team to ensure consensus about the extracted data or considering information as missing. Moreover, dependencies in the data arose from multiple studies (often secondary analyses) being conducted on the same or overlapping samples. We therefore repeated the analyses on independent data only. Further, we cannot be certain that our classifications of study populations, designs, and PROMs represent the best characteristics to highlight heterogeneity. A more important caveat is related to a recent review of response shift methods [8], which concluded that for each response shift method extra steps need to be taken to ensure that the results can indeed be qualified as response shift, i.e., the effects need to be caused by change in meaning of the self-report (see also Vanier et al. [17] and Sprangers et al. [29]). In the present study, we considered all detected effects as response shift effects, although their substantiation may be questioned. However, this limitation is inherent to the current stage of response shift research rather than this systematic review. Further, the heterogeneity of the results may, in part, be due to the variety of detection methods. However, this systematic review cannot disentangle the heterogeneity induced by variation of methods, study context, and design. Another limitation is that the use of different methods and metrics may preclude a clear view of how the resulting numbers compare. We therefore have provided a table where each method is described, response shift detection is detailed, the two most prevalent methods (then-test and Oort’s SEM approach) are further explained, and the effect size metric for all applicable methods are provided (see Table 1). We would like to add that we provided an overview of the various methods and metrics as intended and did not aim to solve the inherent heterogeneity of response shift research. We acknowledge the plea for conceptual and operational clarity of what response shift is, but this is beyond the scope of this systematic review. We also would like to highlight that such a plea is not limited to response shift, but equally applicable to the quality-of-life research field at large and for that matter also to other behavioral and social science research [30]. Further, we did not perform an assessment of methodological quality of individual studies. The heterogeneity of the included studies with regard to response shift methods, population characteristics, study design, and PROMs used precludes such an unambiguous assessment. For example, sample size does not apply as a quality criterion to individual methods. Rather than weighing different study aspects as an indication of study quality, we made them the focus of our main analyses, by describing the prevalence and, where possible, the magnitude of response shift effects for each response shift method, population, study designs, and PROM characteristic. Finally, whereas this descriptive review provides insight into how response shift effects and effect sizes vary per characteristic, it does not allow for direct comparison of effects across characteristics, however, tempting. Studies examining the same characteristic may differ in many other relevant aspects. For example, the number of response shift parameters in a SEM model is a multitude of those of other methods (e.g., the then-test). Moreover, latent-variable methods can only detect response shift when it affects a minority of items and a majority of study participants [31]. Hence, the percentage of detected response shift effects is generally lower and not directly comparable to other methods. Moreover, response shift effects or effect sizes are based on different numbers of studies. Generally, more extreme numbers were found for methods and characteristics based on fewer studies or response shift effects. We therefore only described the results of recalibration response shift when they were based on at least 10 studies and 100 effects. This arbitrary cut-off was intended to guard against over-interpretation of the results. In the case of reprioritization and reconceptualization response shift, the small number of effects reported precluded the application of such criteria. We also refrained from using qualifiers as a higher or lower prevalence and magnitude of response shift effects and provided the minimum and maximum numbers instead.

The current descriptive review on results of quantitative response shift studies is the most comprehensive to date. The data provide insight into the heterogeneity of response shift results, i.e., how the number and magnitude of response shift effects differ across studies employing different response shift methods, populations, research designs, and PROMs. In this sense, this paper draws attention to what some scholars may find a foundational issue in response shift research—the longstanding challenge to harmonize different metrics of response shift across the various measurement procedures from which it is derived. But even in the absence of such harmonization, insight into response shift effects and effect sizes can inform future planning of longitudinal PROM studies, guide the selection of the requisite PROM(s), provide important information for analyzing PROM data in diverse populations, and most importantly, will identify those respondents susceptible to response shift effects for whom different healthcare decision may need to be made.

### Supplementary Information

Below is the link to the electronic supplementary material.

Supplementary file1 (PDF 353 kb)

Supplementary file2 (PDF 453 kb)

## Data Availability

A data file including all data reported in this manuscript can be obtained by contacting the corresponding author.
