# Supplementary material for: Response shift results of quantitative research using patient-reported outcome measures: a descriptive systematic review
Source: Qual Life Res. 2023 Sep 13;33(2):293–315. doi: 10.1007/s11136-023-03495-x (PMC10850024; doi:10.1007/s11136-023-03495-x)
Supplement: Supplementary file 1 — Supplementary file1 (PDF 353 kb) [file 11136_2023_3495_MOESM1_ESM.pdf]

Response shift synthesis – Supplementary Tables of Unrelated Studies and Independent Samples

Table S1: Prevalence of response-shift results by method, based on unrelated studies and independent samples

	Study-level results		Effect-level results					
	Total effects		Total effects		Recalibration		Reprioritization and/or Reconceptualization, or Unknown[a]	
	N	% RS detected	N	% RS detected	N	% RS detected	N	% RS detected
**Design-based methods:**								
Then-test	74	85.1	565	48.3	565	48.3	0	0.0
Individualized methods	9	100.0	19	84.2	2	100.0	17	82.4
Other	11	72.7	109	20.2	12	50.0	97	16.5
**Latent variable models:**								
SEM	36	83.3	2467	8.7	812	17.5	1655	4.4
IRT/Rasch	2	100	51	11.8	46	4.3	5	80.0
**Regression methods:**								
With classification	9	77.8	37	78.4	8	100.0	29	72.4
Without classification	11	81.8	327	14.4	5	40.0	322	14.0
**Other study-specific methods**	4	50.0	4	50.0	0	0.0	4	50.0
Total	134	84	3579	17.0	1450	30.0	2129	8.2

Note. [a)]**Unknown** includes several effects for which the pathway was unknown due to it not being explicitly reported. **Unrelated studies** are based on samples that do not overlap with samples from other studies. When studies are related, only the first (original) study is counted. **Independent samples** do not have overlap with other samples within or across studies. When samples are overlapping, only the overall sample is counted (subsamples are not counted). N = the number of studies or response shift effects. For each study, response shift methods were only counted when results about the response shift effects were reported. For studies that reported the same results in multiple manuscripts, only the results of the first published study were counted. % RS detected = the percentage of detected response shift effects of # studies or # response shift effects that were investigated or possible. SEM = Structural Equation Model. IRT/Rasch = Item Response Theory / Rasch Measurement Model. n/a = not applicable.

Table S2: Prevalence and magnitude of effect-sizes by method, based on unrelated studies and independent samples

	Study-level results		Effect-level results							
	Total effects		Total effects		Recalibration effects			Reprioritization and/or Reconceptualization effects, or Unknown[a]		
	Prevalence		Prevalence		Prevalence		Magnitude	Prevalence		Magnitude
**RS Metric and Method**	N	% RS detected	N	% RS detected	N	% RS detected	ES	N	% RS detected	ES
**Cohen's *d*[b]**	**78**	**87.2**	**612**	**59.0**			**median (IQR)**			**median (IQR)**
Design-based methods: then-test	64	85.9	500	52.2	500	52.2	0.21 (0.10-0.39)	n/a	n/a	n/a
Design-based methods: other	3	66.7	15	46.7	4	50.0	0.28 (0.09-0.45)	11	45.5	0.09 (0.05-0.17)
Latent variable models: SEM	14	100.0	97	95.9	72	100.0	0.24 (0.17-0.37)	25	84.0	0.08 (0.01-0.14)
**R-squared: median (min-max)**	**2**	**100.0**	**27**	**25.9**			**median (IQR)**			**median (min-max)**
Regression without classification	2	100.0	27	25.9	0	n/a	n/a	27	25.9	0.01 (0.00-0.02)
**Classification: % respondents with RS**	**13**	**100.0**	**15**	**100.0**			**people with RS**			**people with RS**
Design-based methods: Individualized	6	100.0	8	100.0	0	n/a	n/a	8	100.0	72.8
Design-based methods: Other	1	100.0	1	100.0	0	n/a	n/a	1	100.0	48.6
Regression methods with classification	4	100.0	4	100.0	2	100.0	n/a	2	100.0	33.8
Other study-specific methods	2	100.0	2	100.0	0	n/a	n/a	2	100.0	13.3
**Other Effect Size metric**	**3**	**66.7**	**5**	**80.0**						
Design-based methods: Individualized	1	100.0	1	100.0	1	100.0	n/a	0	n/a	n/a
Latent variable models: SEM	1	100.0	3	100.0	2	100.0	n/a	1	100.0	n/a
Other study-specific methods	1	0.0	1	0.0	0	n/a	n/a	1	0.0	n/a

Note. Based on a total of 90 unrelated studies and 659 effects from independent samples for which effect sizes could be determined. N = the corresponding # of studies or response shift effects (which are the same for prevalence and magnitudes of the effects). The # of studies (first column) do not add up to 90 or to their respective subtotals (bolded rows) because some studies applied more than one response shift method. % RS detected = the percentage of detected response shift effects of # studies or # response shift effects. % sample = percentage of the pooled sample size (total number of people across studies). SEM = Structural Equation Model. n/a = not applicable because either the response shift pathway could not be investigated due to the method used, or the results could not be discerned from what was reported in the manuscript. IQR = interquartile range. a)**Unknown** includes several effects for which the pathway was unknown due to it not being explicitly reported. b)**Cohen's *d*** was calculated for 951 of the 1062 effects based on the statistical information reported following the procedures described in Table 1. The remaining 111 effects could not be calculated due to inadequate statistical information, in which case we relied on the Cohen's d effect sizes reported in the manuscript (of these 89 effect sizes were explicitly reported as standardized mean differences and 22 effect sizes were assumed to be standardize mean differences based on overall description of the methods). R-squared median (min-max) = 50th percentile, minimum and maximum of the R-squared values.

Table S3: Prevalence and magnitude of response shift results across population characteristics, based on unrelated studies and independent samples

	Study-level results		Effect-level results									
	Total effects		Total effects		Recalibration effects				Reprioritization and/or Reconceptualization effects, or Unknown[a]			
	Prevalence[b]		Prevalence[b]		Prevalence[b]		Magnitude[c]		Prevalence[c]		Magnitude[c]	
**Population characteristics**	N	% RS detected	N	% RS detected	N	% RS detected	N	Median ES (IQR)	N	% RS detected	N	Median ES (IQR)
**Sex**												
Mixed	110	82.7	3074	14.9	1164	26.8	101	0.16 (0.09-0.23)	1910	7.6	30	0.09 (0.01-0.17)
Only female	9	100.0	226	36.3	161	43.5	67	0.29 (0.16-0.49)	65	18.5	n/a	n/a
Only male	10	80.0	161	30.4	97	43.3	14	0.37 (0.18-0.66)	64	10.9	6	0.12 (0.05-0.25)
Other/Unknown	5	100.0	118	16.9	28	39.3	576	0.22 (0.11-0.39)	90	10.0	n/a	n/a
**Age**												
Mostly Adults	86	82.6	2248	18.5	909	29.8	105	0.23 (0.09-0.44)	1339	10.8	15	0.14 (0.08-0.25)
Mostly Older Adults	29	86.2	466	22.1	214	36.9	26	0.02 (0.00-0.12)	252	9.5	16	0.05 (0.02-0.12)
Mostly Children/Adolescer	8	87.5	225	12.0	91	29.7	58	0.28 (0.20-0.53)	134	0.0	n/a	n/a
Other/Unknown	11	90.9	640	9.8	236	24.6	576	0.22 (0.11-0.39)	404	1.2	5	0.03 (0.01-0.06)
**Medical condition**												
No	9	88.9	117	21.4	47	38.3	290	0.23 (0.11-0.37)	70	10.0	7	0.05 (0.04-0.09)
Yes: Cancer	38	92.1	1334	17.8	637	31.9	77	0.43 (0.23-0.82)	697	5.0	12	0.04 (0.01-0.13)
Yes: Orthopedic	9	77.8	78	52.6	77	51.9	7	0.20 (0.18-0.29)	1	100.0	n/a	n/a
Yes: Stroke	7	85.7	292	9.9	81	13.6	n/a	n/a	211	8.5	4	0.08 (0.05-0.12)
Yes: Mental Health	8	75.0	442	6.1	149	10.7	177	0.18 (0.09-0.31)	293	3.8	0	n/a
Yes: Other	63	81.0	1316	18.9	459	32.0	576	0.22 (0.11-0.39)	857	11.9	13	0.17 (0.08-0.25)
**Intervention**												
No/Unclear	45	80.0	1271	12.7	394	21.3	361	0.26 (0.14-0.44)	877	8.9	13	0.09 (0.05-0.14)
Yes: Medical	61	86.9	1693	20.7%	784	35.3%	54	0.22 (0.09-0.38)	909	8.1%	15	0.02 (0.00-0.11)
Yes: Psychological	16	93.8	491	10.8%	202	19.3%	49	0.16 (0.07-0.28)	289	4.8%	n/a	n/a
Yes: Other/Unspecified	12	75.0	124	34.7%	70	50.0%	n/a	n/a	54	14.8%	8	0.20 (0.10-0.39)

Note. [a)]**Unknown** includes several effects for which the pathway was unknown due to it not being explicitly reported. [b)]**Prevalence** is based on a total of 134 unrelated studies and 3579 effects from independent samples that were investigated or possible. N = # of studies or response shift effects. The # of studies may not add up to 134 (first column) because some studies implemented multiple methods or had multiple independent samples that were counted separately. % RS detected = the percentage of detected response shift effects of # studies conducted or # response shift effects that were investigated or possible. [c)]**Magnitude** is based on 78 unrelated studies and 612 effects from independent samples for which Cohen's *d* could be determined. N = the # of these response shift effects. ES = Cohen's *d.* IQR = interquartile range. n/a = not applicable because either the response shift pathway could not be investigated due to the method used or the results could not be discerned from what was reported in the manuscript.

Table S4: Prevalence and magnitude of response shift results for different study design characteristics, based on unrelated studies and independent samples

	Study-level results		Effect-level results: Total effects		Recalibration effects				Reprioritization and/or Reconceptualization effects, or Unknown[a]			
	Prevalence[b]		Prevalence[b]		Prevalence[b]		Magnitude[c]		Prevalence[c]		Magnitude[c]	
**Study design characteristics**	N	% RS detected	N	% RS detected	N	% RS detected	N	Median ES (IQR)	N	% RS detected	N	Median ES (IQR)
**Design**												
Observational	111	86.5	2775	17.5	1114	31.0	441	0.20 (0.10-0.38)	1661	8.4	31	0.10 (0.04-0.20)
Experimental	23	73.9	804	15.4	336	26.8	135	0.26 (0.16-0.41)	468	7.3	5	0.03 (0.01-0.06)
**Data analysis**												
Primary analysis	83	85.5	1036	35.0	664	46.5	489	0.22 (0.11-0.39)	372	14.5	15	0.14 (0.08-0.25)
Secondary analysis	50	82.0	2533	9.7	786	16.0	87	0.23 (0.12-0.48)	1747	6.8	21	0.05 (0.02-0.10)
Unknown	1	100.0	10	10.0	n/a	n/a	n/a	n/a	10	10.0	n/a	n/a
**Sample sizes (Binned)**												
Q1 (< 57)	44	77.3	913	14.3	402	22.9	212	0.25 (0.12-0.43)	511	7.6	5	0.03 (0.01-0.06)
Q2 (57 - 254)	61	83.6	846	27.0	419	39.9	210	0.25 (0.12-0.46)	427	14.3	11	0.14 (0.00-0.22)
Q3 (255 - 410)	28	92.9	1171	15.1	393	32.6	121	0.16 (0.09-0.25)	778	6.3	8	0.10 (0.07-0.35)
Q4 (>411)	23	73.9	649	11.2	236	20.3	33	0.24 (0.17-0.42)	413	6.1	12	0.05 (0.02-0.13)
**Time period classification**												
< 1 month	12	75.0	414	14.0	179	25.7	69	0.25 (0.16-0.35)	235	5.1	7	0.01 (0.00-0.03)
1-6 months	21	90.5	255	30.2	83	51.8	44	0.41 (0.23-0.78)	172	19.8	11	0.05 (0.04-0.10)
>6 months - 12 months	32	71.9	646	12.4	257	20.6	64	0.19 (0.09-0.40)	389	6.9	n/a	n/a
> 12 months	76	85.5	1825	18.6	724	33.8	335	0.21 (0.10-0.42)	1101	8.5	14	0.14 (0.08-0.31)
Not reported	12	83.3	439	12.5	207	23.2	64	0.16 (0.10-0.24)	232	3.0	4	0.18 (0.13-0.24)

Note. a)**Unknown** includes several effects for which the pathway was unknown due to it not being explicitly reported. b)**Prevalence** is based on a total of 134 unrelated studies and 3579 effects from independent samples that were investigated or possible. N = # of studies or response shift effects. The # of studies may not add up to 134 (first column) because some studies implemented multiple methods or had multiple independent samples that were counted separately. % RS detected = the percentage of detected response shift effects of # studies conducted or # response shift effects that were investigated or possible. c)**Magnitude** is based on 78 unrelated studies and 612 effects from independent samples for which Cohen's *d* could be determined. N = the # of these response shift effects. ES = Cohen's *d.* IQR = interquartile range. n/a = not applicable because either the response shift pathway could not be investigated due to the method used or the results could not be discerned from what was reported in the manuscript.

Table S5: Prevalence and magnitude of response shift results for different PROM categories, based on unrelated studies and independent samples

			Effect-level results									
	Study-level results		Total effects		Recalibration effects				Reprioritization and/or Reconceptualization effects, or Unknown[a]			
	Prevalence[a]		Prevalence[a]		Prevalence[a]		Magnitude[b]		Prevalence[a]		Magnitude[b]	
**PROMs**	N	% RS detected	N	% RS detected	N	% RS detected	N	ES (IQR)	N	% RS detected	N	ES (IQR)
**PROM types**												
**Generic PROMs**[a]	**65**	83.1	1598	14.5	588	24.5	210	0.23 (0.10-0.44)	1010	8.7	16	0.08 (0.01-0.17)
#1 SF family[b]	**38**	86.8	1020	16.4	336	26.2	134	0.29 (0.12-0.51)	684	11.5	16	0.08 (0.01-0.17)
#2 EQ 5D[b]	**12**	75.0	40	30.0	24	33.3	20	0.10 (0.04-0.35)	16	25.0	n/a	n/a
#3 Other	**21**	76.2	538	9.9	228	21.1	56	0.22 (0.06-0.34)	310	1.6	n/a	n/a
**Disease-Specific PROMs**												
#1 EORTC family[b]	**15**	86.7	330	27.0	208	37.0	119	0.14 (0.08-0.24)	122	9.8	4	0.17 (0.09-0.47)
#2 WOMAC	**4**	50.0	9	55.6	9	55.6	8	0.20 (0.14-0.29)	n/a	n/a	n/a	n/a
#3 Other	**36**	75.0	691	19.0	294	34.4	115	0.27 (0.16-0.46)	397	7.6	n/a	n/a
**Individualized PROM**	**8**	100.0	25	84.0	8	87.5	6	0.23 (0.20-0.30)	17	82.4	n/a	n/a
**Other type of PROM**	**40**	70.0	926	14.1	343	29.4	154	0.25 (0.14-0.38)	583	5.1	16	0.07 (0.03-0.14)
**PROM domains**												
General health/QOL	**84**	65.5	385	26.2	178	33.7	93	0.24 (0.08-0.42)	207	19.8	2	0.17 (0.14-0.20)
Physical	**85**	69.4	1154	18.5	491	32.8	235	0.21 (0.12-0.35)	663	7.8	16	0.08 (0.02-0.28)
Psychological: depression	**8**	62.5	145	11.7	50	32.0	4	0.21 (0.16-0.26)	95	1.1	n/a	n/a
Psychological: other	**76**	60.5	878	13.9	334	24.9	130	0.19 (0.08-0.31)	544	7.2	12	0.09 (0.03-0.13)
Social	**50**	60.0	470	12.3	175	23.4	53	0.20 (0.13-0.38)	295	5.8	4	0.08 (0.03-0.18)
Pain	**39**	66.7	193	29.5	80	61.3	55	0.39 (0.21-0.55)	113	7.1	n/a	n/a
Other	**28**	53.6	354	11.6	142	17.6	42	0.23 (0.10-0.35)	212	7.5	2	0.05 (0.00-0.09)

Note. [a)]**Unknown** includes several effects for which the pathway was unknown due to it not being explicitly reported. [b)]**Prevalence** is based on a total of 134 unrelated studies and 3579 effects from independent samples that were investigated or possible. N = # of studies or response shift effects. The # of studies may not add up to 134 (first column) because some studies implemented multiple methods or had multiple independent samples that were counted separately. % RS detected = the percentage of detected response shift effects of # studies conducted or # response shift effects that were investigated or possible. [c)]**Magnitude** is based on 76 unrelated studies and 604 effects from independent samples for which Cohen's *d* could be determined. N = the # of these response shift effects. ES = Cohen's *d.* IQR = interquartile range. n/a = not applicable because either the response shift pathway could not be investigated due to the method used or the results could not be discerned from what was reported in the manuscript. SF = Short Form Health Survey, EQ-5D = EuroQol 5 Dimensions, EORTC = European Organisation for Research and Treatment of Cancer.
[a]Bolded values indicate pooled results for all generic or disease-specific PROMs. [b] First and second most frequent PROM based on the # of independent studies in which the PROM was used (the # of effects were used when two PROMs are tied based on the # of studies alone).
