# Supplementary material for: Response shift results of quantitative research using patient-reported outcome measures: a descriptive systematic review
Source: Qual Life Res. 2023 Sep 13;33(2):293–315. doi: 10.1007/s11136-023-03495-x (PMC10850024; doi:10.1007/s11136-023-03495-x)
Supplement: Supplementary file 2 — Supplementary file2 (PDF 453 kb) [file 11136_2023_3495_MOESM2_ESM.pdf]

Appendix: Reference list of included studies (Secondary related studies are shaded)

1. Abolhassani, N., Santos-Eggimann, B., Bula, C., Goy, R., Guessous, I., & Henchoz, Y. (2019). Temporal changes in importance of quality of life domains: a longitudinal study in community-dwelling Swiss older people. Qual Life Res, 28(2), 421-428.
2. Aburub, A. S., Gagnon, B., Ahmed, S., Rodríguez, A. M., & Mayo, N. E. (2018). Impact of reconceptualization response shift on rating of quality of life over time among people with advanced cancer. Supportive Care in Cancer, 26(9), 3063-3071.
3. Addington-Hall, J., Hunt, K., Rowsell, A., Heal, R., Hansford, P., Monroe, B., & Sykes, N. (2014). Development and initial validation of a new outcome measure for hospice and palliative care: the St Christopher's Index of Patient Priorities (SKIPP). BMJ Support Palliat Care, 4(2), 175-181.
4. Ahmed, S., Bourbeau, J., Maltais, F., & Mansour, A. (2009). The Oort structural equation modeling approach detected a response shift after a COPD self-management program not detected by the Schmitt technique. Journal of Clinical Epidemiology, 62(11), 1165-1172.
5. Ahmed, S., Mayo, N., Scott, S., Kuspinar, A., & Schwartz, C. (2011). Using latent trajectory analysis of residuals to detect response shift in general health among patients with multiple sclerosis article. Quality of Life Research, 20(10), 1555-1560.
6. Ahmed, S., Mayo, N. E., Corbiere, M., Wood-Dauphinee, S., Hanley, J., & Cohen, R. (2005). Change in quality of life of people with stroke over time: true change or response shift? Qual Life Res, 14(3), 611-627.
7. Ahmed, S., Mayo, N. E., Wood-Dauphinee, S., Hanley, J. A., & Cohen, S. R. (2004). Response shift influenced estimates of change in health-related quality of life poststroke. J Clin Epidemiol, 57(6), 561-570.
8. Ahmed, S., Mayo Nancy, E., Wood-Dauphinee, S., Hanley James, A., & Cohen, S. R. (2005). The structural equation modeling technique did not show a response shift, contrary to the results of the then test and the individualized approaches. Journal of clinical epidemiology, 58(11), 1125-1133.
9. Ahmed, S., Mayo Nancy, E., Wood-Dauphinee, S., Hanley James, A., & Cohen, S. R. (2005). Using the Patient Generated Index to evaluate response shift post-stroke. Quality of life research : an international journal of quality of life aspects of treatment, care and rehabilitation, 14(10), 2247-2257.
10. Ahmed, S., Sawatzky, R., Levesque, J. F., Ehrmann-Feldman, D., & Schwartz, C. E. (2014). Minimal evidence of response shift in the absence of a catalyst. Qual Life Res, 23(9), 2421-2430.
11. Andrykowski, M. A., Donovan, K. A., & Jacobsen, P. B. (2009). Magnitude and Correlates of Response Shift in Fatigue Ratings in Women Undergoing Adjuvant Therapy for Breast Cancer. Journal of Pain and Symptom Management, 37(3), 341-351.
12. Anota, A., Bascoul-Mollevi, C., Conroy, T., Guillemin, F., Velten, M., Jolly, D., Mercier, M., Causeret, S., Cuisenier, J., Graesslin, O., Hamidou, Z., & Bonnetain, F. (2014). Item response theory and factor analysis as a mean to characterize occurrence of response shift in a longitudinal quality of life study in breast cancer patients. Health and Quality of Life Outcomes, 12(1), 32.
13. Arthur, J., Watts, T., Davies, R., Manchaiah, V., & Slater, J. (2016). An Exploratory Study Identifying a Possible Response Shift Phenomena of the Glasgow Hearing Aid Benefit Profile. Audiology Research, 6(2), 44-48.
14. Balain, B., Ennis, O., Kanes, G., Singhal, R., Roberts, S. N., Rees, D., & Kuiper, J. H. (2009). Response shift in self-reported functional scores after knee microfracture for full thickness cartilage lesions. Osteoarthritis Cartilage, 17(8), 1009-1013.

15. Bar-On, D., Lazar, A., & Amir, M. (2000). Quantitative assessment of response shift in QOL research. Social Indicators Research, 49(1), 37-49.
16. Barclay, R., & Tate, R. B. (2014). Response shift recalibration and reprioritization in health-related quality of life was identified prospectively in older men with and without stroke. Journal of Clinical Epidemiology, 67(5), 500-507.
17. Barclay-Goddard, R., Lix, L. M., Tate, R., Weinberg, L., & Mayo, N. E. (2009). Response shift was identified over multiple occasions with a structural equation modeling framework. Journal of Clinical Epidemiology, 62(11), 1181-1188.
18. Barclay-Goddard, R., Lix, L. M., Tate, R., Weinberg, L., & Mayo, N. E. (2011). Health-Related Quality of Life After Stroke: Does Response Shift Occur in Self-Perceived Physical Function? Archives of Physical Medicine and Rehabilitation, 92(11), 1762-1769.
19. Bastianelli, A., Gius, E., & Cipolletta, S. (2016). Changes over time in the quality of life, prolonged grief and family strain of family caregivers of patients in vegetative state: A pilot study. J Health Psychol, 21(5), 844-852.
20. Bernhard, J., Hürny, C., Maibach, R., Herrmann, R., & Laffer, U. (1999). Quality of life as subjective experience: Reframing of perception in patients with colon cancer undergoing radical resection with or without adjuvant chemotherapy. Annals of Oncology, 10(7), 775-782.
21. Bernhard, J., Lowy, A., Maibach, R., & Hurny, C. (2001). Response shift in the perception of health for utility evaluation. an explorative investigation. Eur J Cancer, 37(14), 1729-1735.
22. Bernhard, J., Lowy, A., Mathys, N., Herrmann, R., & Hurny, C. (2004). Health related quality of life: a changing construct? Qual Life Res, 13(7), 1187-1197.
23. Blanchin, M., Sébille, V., Guilleux, A., & Hardouin, J.-B. (2016). The Guttman errors as a tool for response shift detection at subgroup and item levels. Quality of Life Research, 25(6), 1385-1393.
24. Boucekine, M., Boyer, L., Baumstarck, K., Millier, A., Ghattas, B., Auquier, P., & Toumi, M. (2015). Exploring the response shift effect on the quality of life of patients with schizophrenia: an application of the random forest method. Med Decis Making, 35(3), 388-397.
25. Boucekine, M., Loundou, A., Baumstarck, K., Minaya-Flores, P., Pelletier, J., Ghattas, B., & Auquier, P. (2013). Using the random forest method to detect a response shift in the quality of life of multiple sclerosis patients: a cohort study. BMC Medical Research Methodology, 13(1), 20.
26. Brinksma, A., Tissing, W. J., Sulkers, E., Kamps, W. A., Roodbol, P. F., & Sanderman, R. (2014). Exploring the response shift phenomenon in childhood patients with cancer and its effect on health-related quality of life. Oncol Nurs Forum, 41(1), 48-56.
27. Broberger, E., Sprangers, M., & Tishelman, C. (2006). Do internal standards of quality of life change in lung cancer patients? Nursing research, 55(4), 274-282.
28. Brook, J., Akin, B. A., Lloyd, M., Bhattarai, J., & McDonald, T. P. (2016). The Use of Prospective Versus Retrospective Pretests with Child-Welfare Involved Families. Journal of Child and Family Studies, 25(9), 2740-2752.
29. Chen, P. Y., & Yang, C. M. (2020). Consequences of ignoring the response-shift and measure non-invariant items in sleep studies: an empirical data based simulation of the treatment effect of CBT-I on dysfunctional sleep beliefs. Sleep Med, 74, 99-108.
30. Chen, P.-Y., Jan, Y.-W., & Yang, C.-M. (2017). Are the Insomnia Severity Index and Pittsburgh Sleep Quality Index valid outcome measures for Cognitive Behavioral Therapy for Insomnia? Inquiry from the perspective of response shifts and longitudinal measurement invariance in their Chinese versions. Sleep Medicine, 35, 35-40.
31. Chin, K., Fukuhara, S., Takahashi, K., Sumi, K., Matsumoto, H., Niimi, A., Hattori, N., Mishima, M., & Nakamura, T. (2004). Response shift in perception of sleepiness in obstructive sleep apnea-hypopnea syndrome before and after treatment with nasal CPAP. Sleep, 27(3), 490-493.

32. Chow, E., Chiu, H., Doyle, M., Hruby, G., Holden, L., Barnes, E. A., Tsao, M., Mallia, G., Harris, K., & Danjoux, C. (2007). Patient expectation of the partial response and response shift in pain score. Support Cancer Ther, 4(2), 110-118.
33. Dabakuyo, T. S., Guillemin, F., Conroy, T., Velten, M., Jolly, D., Mercier, M., Causeret, S., Cuisenier, J., Graesslin, O., Gauthier, M., & Bonnetain, F. (2012). Response shift effects on measuring post-operative quality of life among breast cancer patients: a multicenter cohort study. Quality of Life Research, 22(1), 1-11.
34. de Beurs, D. P., Fokkema, M., de Groot, M. H., de Keijser, J., & Kerkhof, A. J. (2015). Longitudinal measurement invariance of the Beck Scale for Suicide Ideation. Psychiatry Res, 225(3), 368-373.
35. DeConde, A. S., Bodner, T. E., Mace, J. C., & Smith, T. L. (2014). Response Shift in Quality of Life After Endoscopic Sinus Surgery for Chronic Rhinosinusitis. JAMA Otolaryngology–Head & Neck Surgery, 140(8), 712-719.
36. Dempster, M., Carney, R., & McClements, R. (2010). Response shift in the assessment of quality of life among people attending cardiac rehabilitation. British Journal of Health Psychology, 15(2), 307-319.
37. Echteld, M. A., van Zuylen, L., Bannink, M., Witkamp, E., & Van der Rijt, C. C. (2007). Changes in and correlates of individual quality of life in advanced cancer patients admitted to an academic unit for palliative care. Palliat Med, 21(3), 199-205.
38. Echteld Michael, A., Deliens, L., Ooms Marcel, E., Ribbe Miel, W., van der, W., & Gerrit. (2005). Quality of life change and response shift in patients admitted to palliative care units: a pilot study. Palliative medicine, 19(5), 381-388.
39. Edelaar-Peeters, Y., & Stiggelbout, A. M. (2013). Anticipated adaptation or scale recalibration? Health Qual Life Outcomes, 11, 171.
40. Felix, J., Becker, C., Vogl, M., Buschner, P., Plotz, W., & Leidl, R. (2019). Patient characteristics and valuation changes impact quality of life and satisfaction in total knee arthroplasty - results from a German prospective cohort study. Health Qual Life Outcomes, 17(1), 180.
41. Serdà i Ferrer, B.-C., Valle, A. d., & Marcos-Gragera, R. (2014). Prostate Cancer and Quality of Life: Analysis of Response Shift Using Triangulation Between Methods. Journal of Gerontological Nursing, 40(6), 32-41.
42. Finkelstein, J. A., Quaranto, B. R., & Schwartz, C. E. (2013). Threats to the Internal Validity of Spinal Surgery Outcome Assessment: Recalibration Response Shift or Implicit Theories of Change? Applied Research in Quality of Life, 9(2), 215-232.
43. Friedrich, M., Karoff, J., & Hinz, A. (2019). Response shift effects in patients' assessments of their quality of life after cardiac rehabilitation. Quality of Life Research, 28(9), 2609-2620.
44. Friedrich, M., Zenger, M., & Hinz, A. (2019). Response shift effects of quality of life assessments in breast cancer survivors. Eur J Cancer Care (Engl), 28(2), e12979.
45. Gadermann, A. M., Sawatzky, R., Palepu, A., Hubley, A. M., Zumbo, B. D., Aubry, T., Farrell, S., & Hwang, S. W. (2017). Minimal impact of response shift for SF-12 mental and physical health status in homeless and vulnerably housed individuals: an item-level multi-group analysis. Qual Life Res, 26(6), 1463-1472.
46. Galenkamp, H., Huisman, M., Braam, A. W., & Deeg, D. J. (2012). Estimates of prospective change in self-rated health in older people were biased owing to potential recalibration response shift. Journal of Clinical Epidemiology, 65(9), 978-988.
47. Gandhi, P. K., Schwartz, C. E., Reeve, B. B., DeWalt, D. A., Gross, H. E., & Huang, I. C. (2016). An item-level response shift study on the change of health state with the rating of asthma-specific quality of life: a report from the PROMIS® Pediatric Asthma Study. Quality of Life Research, 25(6), 1349-1359.

48. Gandhi, P. K., Ried, L. D., Huang, I. C., Kimberlin, C. L., & Kauf, T. L. (2012). Assessment of response shift using two structural equation modeling techniques. Quality of Life Research, 22(3), 461-471.
49. Gerlich, C., Schuler, M., Jelitte, M., Neuderth, S., Flentje, M., Graefen, M., Krüger, A., Mehnert, A., & Faller, H. (2016). Prostate cancer patients' quality of life assessments across the primary treatment trajectory: 'True' change or response shift? Acta Oncologica, 55(7), 814-820.
50. Gillison, F., Skevington, S., & Standage, M. (2008). Exploring response shift in the quality of life of healthy adolescents over 1 year. Qual Life Res, 17(7), 997-1008.
51. Guilleux, A., Blanchin, M., Vanier, A., Guillemin, F., Falissard, B., Schwartz, C. E., Hardouin, J. B., & Sebille, V. (2015). RespOnse Shift ALgorithm in Item response theory (ROSALI) for response shift detection with missing data in longitudinal patient-reported outcome studies. Qual Life Res, 24(3), 553-564.
52. Haagsma, J. A., Spronk, I., de Jongh, M. A. C., Bonsel, G. J., & Polinder, S. (2020). Conventional and retrospective change in health-related quality of life of trauma patients: an explorative observational follow-up study. Health and Quality of Life Outcomes, 18(1), 1-13.
53. Hagedoorn, M., Sneeuw, K. C., & Aaronson, N. K. (2002). Changes in physical functioning and quality of life in patients with cancer: response shift and relative evaluation of one's condition. J Clin Epidemiol, 55(2), 176-183.
54. Hamidou, Z., Dabakuyo-Yonli, T. S., Guillemin, F., Conroy, T., Velten, M., Jolly, D., Causeret, S., Graesslin, O., Gauthier, M., Mercier, M., & Bonnetain, F. (2014). Impact of response shift on time to deterioration in quality of life scores in breast cancer patients. PLoS One, 9(5), e96848.
55. Hinz, A., Finck Barboza, C., Zenger, M., Singer, S., Schwalenberg, T., & Stolzenburg, J. U. (2011). Response shift in the assessment of anxiety, depression and perceived health in urologic cancer patients: an individual perspective. Eur J Cancer Care (Engl), 20(5), 601-609.
56. Hinz, A., Karoff, J., Kittel, J., Brähler, E., Zenger, M., Schmalbach, B., & Kocalevent, R.-D. (2020). Associations between self-rated health and the assessments of anchoring vignettes in cardiovascular patients. International Journal of Clinical and Health Psychology, 20(2), 100-107.
57. Höfer, S., Pfaffenberger, N., Renn, D., Platter, M., & Ring, L. (2010). Coronary Intervention Improves Disease Specific Health-Related Quality of Life but Not Individualised Quality of Life: A Potential Response Shift Effect? Applied Research in Quality of Life, 6(1), 81-90.
58. Hollman, F., Wessel, R. N., & Wolterbeek, N. (2016). Response shift of the Western Ontario Rotator Cuff index in patients undergoing arthroscopic rotator cuff repair. J Shoulder Elbow Surg, 25(12), 2011-2018.
59. Hosseini, B., Nedjat, S., Zendehdel, K., Majdzadeh, R., Nourmohammadi, A., & Montazeri, A. (2017). Response shift in quality of life assessment among cancer patients: A study from Iran. Medical Journal of the Islamic Republic of Iran, 31(1), 798-803.
60. Howard, J. S., Mattacola, C. G., Mullineaux, D. R., English, R. A., & Lattermann, C. (2014). Influence of response shift on early patient-reported outcomes following autologous chondrocyte implantation. Knee Surg Sports Traumatol Arthrosc, 22(9), 2163-2171.
61. Inoue, M., Kasai, T., Kawana, F., & Narui, K. (2007). Response shift of subjective sleepiness in patients with obstructive sleep apnea-hypopnea syndrome. Sleep and Biological Rhythms, 5(2), 95-99.
62. Ito, N., Ishiguro, M., Tanaka, M., Tokunaga, K., Sugihara, K., & Kazuma, K. (2010). Response shift in quality-of-life assessment in patients undergoing curative surgery with permanent colostomy: a preliminary study. Gastroenterol Nurs, 33(6), 408-412.
63. Jabrayilov, R., Emons, W. H. M., de Jong, K., & Sijtsma, K. (2017). Longitudinal measurement invariance of the Dutch Outcome Questionnaire-45 in a clinical sample. Qual Life Res, 26(6), 1473-1481.

64. Jakola, A. S., Solheim, O., Gulati, S., & Sagberg, L. M. (2016). Is there a response shift in generic health-related quality of life 6 months after glioma surgery? Acta Neurochirurgica, 159(2), 377-384.
65. Jansen, S. J., Stiggelbout, A. M., Nooij, M. A., Noordijk, E. M., & Kievit, J. (2000). Response shift in quality of life measurement in early-stage breast cancer patients undergoing radiotherapy. Qual Life Res, 9(6), 603-615.
66. Joore, M. A., Potjewijd, J., Timmerman, A. A., & Anteunis, L. J. (2002). Response shift in the measurement of quality of life in hearing impaired adults after hearing aid fitting. Qual Life Res, 11(4), 299-307.
67. Kievit, W., Hendrikx, J., Stalmeier, P. F., van de, L., M, A., Van, R., P, L., & Adang, E. M. (2010). The relationship between change in subjective outcome and change in disease: a potential paradox. Quality of life research : an international journal of quality of life aspects of treatment, care and rehabilitation, 19(7), 985-994.
68. Kimura, A., Arakawa, H., Noda, K., Yamazaki, S., Hara, E. S., Mino, T., Matsuka, Y., Mulligan, R., & Kuboki, T. (2012). Response shift in oral health-related quality of life measurement in patients with partial edentulism. J Oral Rehabil, 39(1), 44-54.
69. King-Kallimanis, B. L., Oort, F. J., & Garst, G. J. A. (2010). Using structural equation modelling to detect measurement bias and response shift in longitudinal data. AStA Advances in Statistical Analysis, 94(2), 139-156.
70. King-Kallimanis, B. L., Oort, F. J., Nolte, S., Schwartz, C. E., & Sprangers, M. A. G. (2011). Using structural equation modeling to detect response shift in performance and health-related quality of life scores of multiple sclerosis patients. Quality of Life Research, 20(10), 1527-1540.
71. King-Kallimanis, B. L., Oort, F. J., Visser, M. R., & Sprangers, M. A. (2009). Structural equation modeling of health-related quality-of-life data illustrates the measurement and conceptual perspectives on response shift. J Clin Epidemiol, 62(11), 1157-1164.
72. Korfage Ida, J., de, K., Harry, J., & Essink-Bot, M.-L. (2007). Response shift due to diagnosis and primary treatment of localized prostate cancer: a then-test and a vignette study. Quality of life research : an international journal of quality of life aspects of treatment, care and rehabilitation, 16(10), 1627-1634.
73. Kubota, Y., Yoneda, K., Nakai, K., Katsuura, J., Moriue, T., Matsuoka, Y., Miyamoto, I., & Ohya, Y. (2009). Effect of sequential applications of topical tacrolimus and topical corticosteroids in the treatment of pediatric atopic dermatitis: an open-label pilot study. J Am Acad Dermatol, 60(2), 212-217.
74. Kuijer, R. G., De Ridder, D. T. D., Colland, V. T., Schreurs, K. M. G., & Sprangers, M. A. G. (2007). Effects of a short self-management intervention for patients with asthma and diabetes: Evaluating health-related quality of life using then-test methodology. Psychology & Health, 22(4), 387-411.
75. Kvam, A. K., Wisløff, F., & Fayers, P. M. (2010). Minimal important differences and response shift in health-related quality of life; a longitudinal study in patients with multiple myeloma. Health and Quality of Life Outcomes, 8(1), 79.
76. Lepore, S. J., & Eton, D. T. (2000). Response shifts in prostate cancer patients: An evaluation of suppressor and buffer models. In Adaptation to changing health: Response shift in quality-of-life research. (pp. 37-51).
77. Li, Y., & Rapkin, B. (2009). Classification and regression tree uncovered hierarchy of psychosocial determinants underlying quality-of-life response shift in HIV/AIDS. J Clin Epidemiol, 62(11), 1138-1147.
78. Li, Y., & Schwartz, C. E. (2011). Data mining for response shift patterns in multiple sclerosis patients using recursive partitioning tree analysis. Quality of Life Research, 20(10), 1543-1553.

79. Liu, J. J., & Davis, G. E. (2015). The significance of response shift in sinus surgery outcomes. Int Forum Allergy Rhinol, 5(1), 55-59.
80. Lix, L. M., Chan, E. K., Sawatzky, R., Sajobi, T. T., Liu, J., Hopman, W., & Mayo, N. (2016). Response shift and disease activity in inflammatory bowel disease. Qual Life Res, 25(7), 1751-1760.
81. Lix, L. M., Sajobi, T. T., Sawatzky, R., Liu, J., Mayo, N. E., Huang, Y., Graff, L. A., Walker, J. R., Ediger, J., Clara, I., Sexton, K., Carr, R., & Bernstein, C. N. (2013). Relative importance measures for reprioritization response shift. Qual Life Res, 22(4), 695-703.
82. Machuca, C., Vettore, M. V., Krasuska, M., Baker, S. R., & Robinson, P. G. (2017). Using classification and regression tree modelling to investigate response shift patterns in dentine hypersensitivity. BMC Medical Research Methodology, 17(1), 120.
83. Machuca, C., Vettore, M. V., & Robinson, P. G. (2020). How peoples' ratings of dental implant treatment change over time? Quality of Life Research, 29(5), 1323-1334.
84. Mayo, N. E., Scott, S. C., Dendukuri, N., Ahmed, S., & Wood-Dauphinee, S. (2008). Identifying response shift statistically at the individual level. Quality of Life Research, 17(4), 627-639.
85. Mayo, N. E., Scott, S. C., & Ahmed, S. (2009). Case management poststroke did not induce response shift: the value of residuals. Journal of Clinical Epidemiology, 62(11), 1148-1156.
86. Mayo, N. E., Scott, S. C., Bernstein, C. N., & Lix, L. M. (2015). How are you? Do people with inflammatory bowel disease experience response shift on this question? Health and Quality of Life Outcomes, 13(1), 52.
87. McPhail, S., & Haines, T. (2010). Response shift, recall bias and their effect on measuring change in health-related quality of life amongst older hospital patients. Health and Quality of Life Outcomes, 8(1), 65-65.
88. Modarresi, S., & Walton, D. M. (2021). Reliability, discriminative accuracy, and an exploration of response shift as measured using the satisfaction and Recovery Index over 12 months from musculoskeletal trauma. Musculoskeletal Science and Practice, 51, 102300.
89. Mollerup, A., & Johansen, J. D. (2015). Response shift in severity assessment of hand eczema with visual analogue scales. Contact Dermatitis, 72(3), 178-183.
90. Murata, T., Suzukamo, Y., Shiroiwa, T., Taira, N., Shimozuma, K., Ohashi, Y., & Mukai, H. (2020). Response Shift-Adjusted Treatment Effect on Health-Related Quality of Life in a Randomized Controlled Trial of Taxane Versus S-1 for Metastatic Breast Cancer: Structural Equation Modeling. Value Health, 23(6), 768-774.
91. Murray, A. L., McKenzie, K., Murray, K., & Richelieu, M. (2018). Examining response shifts in the Clinical Outcomes in Routine Evaluation- Outcome Measure (CORE-OM). British Journal of Guidance & Counselling, 48(2), 276-288.
92. Nagl, M., & Farin, E. (2012). Response shift in quality of life assessment in patients with chronic back pain and chronic ischaemic heart disease. Disabil Rehabil, 34(8), 671-680.
93. Nieuwkerk, P. T., Tollenaar, M. S., Oort, F. J., & Sprangers, M. A. G. (2007). Are Retrospective Measures of Change in Quality of Life More Valid Than Prospective Measures? Medical Care, 45(3), 199-205.
94. Nolte, S., Mierke, A., Fischer, H. F., & Rose, M. (2016). On the validity of measuring change over time in routine clinical assessment: a close examination of item-level response shifts in psychosomatic inpatients. Qual Life Res, 25(6), 1339-1347.
95. Oort Frans, J., Visser Mechteld, R. M., & Sprangers Mirjam, A. G. (2005). An application of structural equation modeling to detect response shifts and true change in quality of life data from cancer patients undergoing invasive surgery. Quality of life research : an international journal of quality of life aspects of treatment, care and rehabilitation, 14(3), 599-609.

96. Ousmen, A., Conroy, T., Guillemin, F., Velten, M., Jolly, D., Mercier, M., Causeret, S., Cuisenier, J., Graesslin, O., Hamidou, Z., Bonnetain, F., & Anota, A. (2016). Impact of the occurrence of a response shift on the determination of the minimal important difference in a health-related quality of life score over time. Health Qual Life Outcomes, 14(1), 167.
97. Persson, L. O., Engstrom, C. P., Ryden, A., Larsson, S., & Sullivan, M. (2005). Life values in patients with COPD: relations with pulmonary functioning and health related quality of life. Qual Life Res, 14(2), 349-359.
98. Postulart, D., & Adang, E. M. M. (2016). Response Shift and Adaptation in Chronically Ill Patients. Medical Decision Making, 20(2), 186-193.
99. Hoch, J. M., Jamali, B. E., Hoch, M. C., & Powden, C. J. (2019). Response Shift After a 4-Week Multimodal Intervention for Chronic Ankle Instability. Journal of Athletic Training, 54(4), 397-402.
100. Powell, G. A., Adair, C. E., Streiner, D. L., Mayo, N., & Latimer, E. (2017). Changes in quality of life from a homelessness intervention: true change, response shift, or random variation. Qual Life Res, 26(7), 1853-1864.
101. Pratt, C. C., McGuigan, W. M., & Katzev, A. R. (2016). Measuring Program Outcomes: Using Retrospective Pretest Methodology. American Journal of Evaluation, 21(3), 341-349.
102. Preiss, M., Friedrich, M., Stolzenburg, J. U., Zenger, M., & Hinz, A. (2019). Response shift effects in the assessment of urologic cancer patients' quality of life. Eur J Cancer Care (Engl), 28(4), e13027.
103. Rapkin Bruce, D. (2000). Personal goals and response shifts: Understanding the impact of illness and events on the quality of life of people living with AIDS. Adaptation to changing health: Response shift in quality-of-life research., 53-71.
104. Razmjou, H., Schwartz, C. E., & Holtby, R. (2010). The impact of response shift on perceived disability two years following rotator cuff surgery. J Bone Joint Surg Am, 92(12), 2178-2186.
105. Razmjou, H., Schwartz, C. E., Yee, A., & Finkelstein, J. A. (2009). Traditional assessment of health outcome following total knee arthroplasty was confounded by response shift phenomenon. Journal of Clinical Epidemiology, 62(1), 91-96.
106. Razmjou, H., Yee, A., Ford, M., & Finkelstein, J. A. (2006). Response shift in outcome assessment in patients undergoing total knee arthroplasty. J Bone Joint Surg Am, 88(12), 2590-2595.
107. Rees, J., Clarke Michael, G., Waldron, D., O'Boyle, C., Ewings, P., & MacDonagh Ruaraidh, P. (2005). The measurement of response shift in patients with advanced prostate cancer and their partners. Health and quality of life outcomes, 3, 21.
108. Rees, J., Waldron, D., O'Boyle, C., Ewings, P., & MacDonagh, R. (2003). Prospective vs retrospective assessment of lower urinary tract symptoms in patients with advanced prostate cancer: the effect of 'response shift'. BJU international, 92(7), 703-706.
109. Reissmann, D. R., Erler, A., Hirsch, C., Sierwald, I., Machuca, C., & Schierz, O. (2017). Bias in retrospective assessment of perceived dental treatment effects when using the Oral Health Impact Profile. Quality of Life Research, 27(3), 775-782.
110. Reissmann, D. R., John, M. T., Feuerstahler, L., Baba, K., Szabó, G., Čelebić, A., & Waller, N. (2016). Longitudinal measurement invariance in prospective oral health-related quality of life assessment. Health and Quality of Life Outcomes, 14(1), 88.
111. Reissmann, D. R., Remmler, A., John, M. T., Schierz, O., & Hirsch, C. (2012). Impact of response shift on the assessment of treatment effects using the Oral Health Impact Profile. European Journal of Oral Sciences, 120(6), 520-525.
112. Ring, L., Hofer, S., Heuston, F., Harris, D., & O'Boyle, C. A. (2005). Response shift masks the treatment impact on patient reported outcomes (PROs): the example of individual quality of life in edentulous patients. Health Qual Life Outcomes, 3, 55.

113. Rutgers, M., Creemers, L. B., Yang, K. G. A., Raijmakers, N. J. H., Dhert, W. J. A., & Saris, D. B. F. (2014). Osteoarthritis treatment using autologous conditioned serum after placebo. Acta Orthopaedica, 86(1), 114-118.
114. Sajobi, T. T., Fiest, K. M., & Wiebe, S. (2014). Changes in quality of life after epilepsy surgery: The role of reprioritization response shift. Epilepsia, 55(9), 1331-1338.
115. Sajobi, T. T., Lix, L. M., Singh, G., Lowerison, M., Engbers, J., & Mayo, N. E. (2015). Identifying reprioritization response shift in a stroke caregiver population: a comparison of missing data methods. Qual Life Res, 24(3), 529-540.
116. Sajobi, T. T., Speechley, K. N., Liang, Z., Goodwin, S. W., Ferro, M. A., & Wiebe, S. (2017). Response shift in parents' assessment of health-related quality of life of children with new-onset epilepsy. Epilepsy & Behavior, 75, 97-101.
117. Salmon, M., Blanchin, M., Rotonda, C., Guillemin, F., & Sébille, V. (2017). Identifying patterns of adaptation in breast cancer patients with cancer-related fatigue using response shift analyses at subgroup level. Cancer Medicine, 6(11), 2562-2575.
118. Schwartz, C. E., Stark, R. B., & Stucky, B. D. (2021). Response-shift effects in neuromyelitis optica spectrum disorder: a secondary analysis of clinical trial data. Qual Life Res, 30(5), 1267-1282.
119. Schwartz, C. E., Stucky, B., Rivers, C. S., Noonan, V. K., & Finkelstein, J. A. (2018). Quality of Life and Adaptation in People With Spinal Cord Injury: Response Shift Effects From 1 to 5 Years Postinjury. Arch Phys Med Rehabil, 99(8), 1599-1608 e1591.
120. Schwartz, C. E., Stucky, B. D., Michael, W., & Rapkin, B. D. (2020). Does response shift impact interpretation of change even among scales developed using item response theory? Journal of Patient-Reported Outcomes, 4(1), 8.
121. Schwartz, C. E., Feinberg, R. G., Jilinskaia, E., & Applegate, J. C. (1999). An evaluation of a psychosocial intervention for survivors of childhood cancer: paradoxical effects of response shift over time. Psychooncology, 8(4), 344-354.
122. Schwartz, C. E., Merriman, M. P., Reed, G. W., & Hammes, B. J. (2004). Measuring patient treatment preferences in end-of-life care research: applications for advance care planning interventions and response shift research. J Palliat Med, 7(2), 233-245.
123. Schwartz, C. E., Powell, V. E., & Rapkin, B. D. (2016). 23rd Annual Conference of the International Society for Quality of Life Research. Quality of Life Research, 25(S1), 1-196.
124. Schwartz, C. E., & Rapkin, B. D. (2012). Understanding appraisal processes underlying the thentest: a mixed methods investigation. Qual Life Res, 21(3), 381-388.
125. Schwartz, C. E., Sajobi, T. T., Lix, L. M., Quaranto, B. R., & Finkelstein, J. A. (2013). Changing values, changing outcomes: the influence of reprioritization response shift on outcome assessment after spine surgery. Qual Life Res, 22(9), 2255-2264.
126. Schwartz, C. E., Sprangers, M. A. G., Carey, A., & Reed, G. (2004). Exploring response shift in longitudinal data. Psychology & Health, 19(1), 51-69.
127. Sharpe, L., Butow, P., Smith, C., McConnell, D., & Clarke, S. (2005). Changes in quality of life in patients with advanced cancer: evidence of response shift and response restriction. J Psychosom Res, 58(6), 497-504.
128. Shi, H. Y., Lee, K. T., Lee, H. H., Uen, Y. H., & Chiu, C. C. (2011). Response shift effect on gastrointestinal quality of life index after laparoscopic cholecystectomy. Qual Life Res, 20(3), 335-341.
129. Smith, D., Woodman, R., Harvey, P., & Battersby, M. (2016). Self-Perceived Distress and Impairment in Problem Gamblers: A Study of Pre- to Post-treatment Measurement Invariance. J Gambl Stud, 32(4), 1065-1078.
130. Sprangers, M. A. (1996). Response-shift bias: a challenge to the assessment of patients' quality of life in cancer clinical trials. Cancer Treat Rev, 22 Suppl A, 55-62.

131. Sprangers Mirjam, A. G., van, D., Frits, S. A. M., Broersen, J., Lodder, L., Wever, L., Visser Mechteld, R. M., Oosterveld, P., & Smets, E. (2000). Response shift and fatigue: The use of the thentest approach. Adaptation to changing health: Response shift in quality-of-life research., 137-151.
132. Spuling, S. M., Wolff, J. K., & Wurm, S. (2017). Response shift in self-rated health after serious health events in old age. Soc Sci Med, 192, 85-93.
133. Berry, D. L., Pett, M. A., Clayton, M. F., Beck, S. L., & Tavernier, S. S. (2011). Validity of the Patient Generated Index as a Quality-of-Life Measure in Radiation Oncology. Oncology Nursing Forum, 38(3), 319-329.
134. ten Ham, R. M. T., Broering, J. M., Cooperberg, M. R., Carroll, P., & Wilson, L. S. (2020). Understanding the Major Factors Affecting Response Shift Effects on Health-Related Quality of Life: What the Then-Test Measures in a Longitudinal Prostate Cancer Registry. Clinical Genitourinary Cancer, 18(1), e21-e27.
135. Tessier, P., Blanchin, M., & Sébille, V. (2017). Does the relationship between health-related quality of life and subjective well-being change over time? An exploratory study among breast cancer patients. Social Science & Medicine, 174, 96-103.
136. Timmerman Angelique, A., Anteunis Lucien, J. C., & Meesters Cor, M. G. (2003). Response-shift bias and parent-reported quality of life in children with otitis media. Archives of otolaryngology--head & neck surgery, 129(9), 987-991.
137. Traa, M. J., Braeken, J., De Vries, J., Roukema, J. A., Orsini, R. G., & Den Oudsten, B. L. (2014). Evaluating quality of life and response shift from a couple-based perspective: a study among patients with colorectal cancer and their partners. Quality of Life Research, 24(6), 1431-1441.
138. van Leeuwen, C. M. C., Post, M. W. M., van der Woude, L. H. V., de Groot, S., Smit, C., van Kuppevelt, D., & Lindeman, E. (2011). Changes in life satisfaction in persons with spinal cord injury during and after inpatient rehabilitation: adaptation or measurement bias? Quality of Life Research, 21(9), 1499-1508.
139. Verdam, M. G. E., Oort, F. J., & Sprangers, M. A. G. (2015). Using structural equation modeling to detect response shifts and true change in discrete variables: an application to the items of the SF-36. Quality of Life Research, 25(6), 1361-1383.
140. Verdam, M. G. E., Oort, F. J., van der Linden, Y. M., & Sprangers, M. A. G. (2014). Taking into account the impact of attrition on the assessment of response shift and true change: a multigroup structural equation modeling approach. Quality of Life Research, 24(3), 541-551.
141. Verdam, M. G. E., Oort, F. J., & Sprangers, M. A. G. (2017). Structural equation modeling–based effect-size indices were used to evaluate and interpret the impact of response shift effects. Journal of Clinical Epidemiology, 85, 37-44.
142. Visser, M. R. M., Oort, F. J., van Lanschot, J. J. B., van der Velden, J., Kloek, J. J., Gouma, D. J., Schwartz, C. E., & Sprangers, M. A. G. (2013). The role of recalibration response shift in explaining bodily pain in cancer patients undergoing invasive surgery: an empirical investigation of the Sprangers and Schwartz model. Psycho-Oncology, 22(3), 515-522.
143. Visser, M. R., Smets, E. M., Sprangers, M. A., de, H., & H, J. (2000). How response shift may affect the measurement of change in fatigue. Journal of pain and symptom management, 20(1), 12-18.
144. Visser, M. R., Oort, F. J., & Sprangers, M. A. (2005). Methods to detect response shift in quality of life data: a convergent validity study. Qual Life Res, 14(3), 629-639.
145. Wagner Julie, A. (2005). Response shift and glycemic control in children with diabetes. Health and quality of life outcomes, 3, 38.

146. Wang, X., Xu, X., Han, H., He, R., Zhou, L., Liang, R., & Yu, H. (2018). Using structural equation modeling to detect response shift in quality of life in patients with Alzheimer's disease. International Psychogeriatrics, 31(1), 123-132.
147. Wu, P. C. (2016). Response Shifts in Depression Intervention for Early Adolescents. J Clin Psychol, 72(7), 663-675.
148. Yang, J., Hanna-Pladdy, B., Gruber-Baldini, A. L., Barr, E., von Coelln, R., Armstrong, M. J., Reich, S. G., & Shulman, L. M. (2017). Response shift - The experience of disease progression in Parkinson disease. Parkinsonism Relat Disord, 36, 52-56.
149. Yardley, L., & Dibb, B. (2007). Assessing subjective change in chronic illness: an examination of response shift in health-related and goal-oriented subjective status. Psychology & Health, 22(7), 813-828.
150. Zhang, X.-H., Li, S.-C., Xie, F., Lo, N.-N., Yang, K.-Y., Yeo, S.-J., Fong, K.-Y., & Thumboo, J. (2012). An Exploratory Study of Response Shift in Health-Related Quality of Life and Utility Assessment Among Patients with Osteoarthritis Undergoing Total Knee Replacement Surgery in a Tertiary Hospital in Singapore. Value in Health, 15(1), S72-S78.
